# *Leishmania major* drives host phagocyte death and cell-to-cell transfer depending on intracellular pathogen proliferation rate

**DOI:** 10.1172/jci.insight.169020

**Published:** 2023-07-24

**Authors:** Iris Baars, Moritz Jaedtka, Leon-Alexander Dewitz, Yan Fu, Tobias Franz, Juliane Mohr, Patricia Gintschel, Hannes Berlin, Angelina Degen, Sandra Freier, Stefan Rygol, Burkhart Schraven, Sascha Kahlfuß, Ger van Zandbergen, Andreas J. Müller

**Affiliations:** 1 Experimental Immunodynamics, Institute of Molecular and Clinical Immunology, Medical Faculty, and; 2 Health Campus Immunology, Infectiology and Inflammation (GCI3), Medical Faculty and Center for Health and Medical Prevention (CHaMP), Otto von Guericke University Magdeburg, Magdeburg, Germany.; 3 Division of Immunology, Paul Ehrlich Institute, Langen, Germany.; 4 Institute for Immunology, University Medical Center, Johannes Gutenberg University Mainz, Mainz, Germany.; 5 Institute of Molecular and Clinical Immunology, Medical Faculty, Otto von Guericke University Magdeburg, Magdeburg, Germany.; 6 Research Center for Immunotherapy (FZI), University Medical Center, Johannes Gutenberg University Mainz, Mainz, Germany.; 7 Helmholtz Centre for Infection Research, Braunschweig, Germany.

**Keywords:** Infectious disease, Microbiology, Apoptosis, Macrophages, Monocytes

## Abstract

The virulence of intracellular pathogens relies largely on the ability to survive and replicate within phagocytes but also on release and transfer into new host cells. Such cell-to-cell transfer could represent a target for counteracting microbial pathogenesis. However, our understanding of the underlying cellular and molecular processes remains woefully insufficient. Using intravital 2-photon microscopy of caspase-3 activation in the *Leishmania major*–infected (*L*. *major*–infected) live skin, we showed increased apoptosis in cells infected by the parasite. Also, transfer of the parasite to new host cells occurred directly without a detectable extracellular state and was associated with concomitant uptake of cellular material from the original host cell. These in vivo findings were fully recapitulated in infections of isolated human phagocytes. Furthermore, we observed that high pathogen proliferation increased cell death in infected cells, and long-term residency within an infected host cell was only possible for slowly proliferating parasites. Our results therefore suggest that *L*. *major* drives its own dissemination to new phagocytes by inducing host cell death in a proliferation-dependent manner.

## Graphical abstract

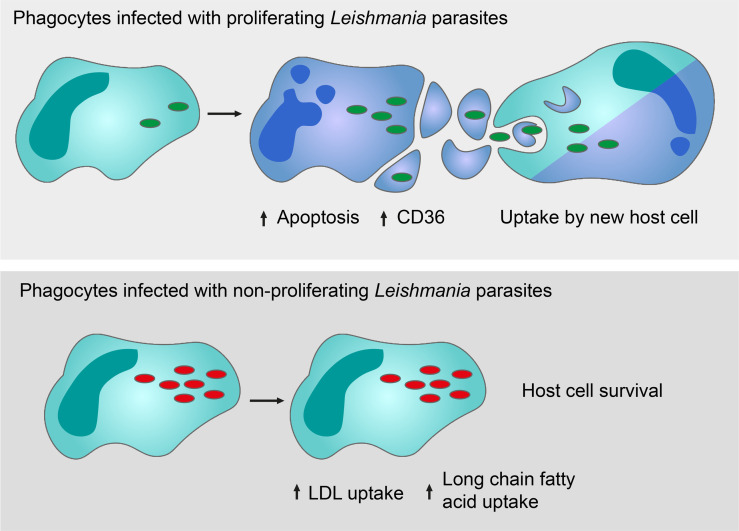

## Introduction

Many pathogens have developed means to evade and subvert the intracellular defense mechanisms of host phagocytes, and their abilities to proliferate within and to spread among these phagocytes are main strategies to establish infection. The exit of such intracellular pathogens from their host cell and subsequent infection of new cells therefore represent a fundamental step in infection and might represent an Achilles’ heel of microbial pathogenesis (1). However, the mechanisms underlying such cell-to-cell transfer remain to be fully elucidated. The parasite *Leishmania major* (*L*. *major*) can survive and replicate under the harsh microenvironmental conditions of endocytic compartments of professional phagocytes, and can establish long-term infection of the skin (2–4). Infectious-stage metacyclic promastigote forms of *L*. *major* are inoculated into the upper dermis by the bite of a sand fly (5). The flagellated promastigotes are rapidly taken up by neutrophils, and a few days after infection, the parasites persist mainly within monocytes, macrophages, and dendritic cells in the form of short, aflagellated amastigotes. These phagocytes have been shown to play a dual role during *Leishmania* infection. On the one hand, in particular, inflammatory monocyte-derived cells have been shown to be important for efficient containment of *L*. *major* through the production of nitric oxide by inducible nitric oxide synthase (iNOS) (2). On the other hand, we have recently shown that CD11c-expressing Ly6C^+^CCR2^+^ monocytes constitute the main reservoir for efficient *Leishmania* proliferation and cell-to-cell transmission (6).

While very early studies in mice postulated that the unrestricted replication of the intracellular amastigotes causes host cell rupture and release of the parasites (7), more recent findings suggested that the release of *Leishmania* from the infected host cells is strictly regulated. For example, Rittig and colleagues found evidence that *L*. *major* is released by murine peritoneal macrophages through exocytosis (8, 9). Furthermore, Noronha and colleagues showed that *L*. *amazonensis* and *L*. *guyanensis* use a pore-forming cytolysin to exit host cells (10–12). Despite these findings, the mechanisms involved in efficient parasite exit and transmission in vivo are still largely unknown. Unraveling these mechanisms is, however, of utmost importance, since *Leishmania* amastigotes, which are proliferating intracellularly, have to leave infected macrophages to infect other cells in order to persist and propagate at the site of infection.

One possible mechanism involved in the spread of *Leishmania* parasites among phagocytic subsets is cell death of the infected host cell. While some studies suggest that *Leishmania* parasites are able to suppress the death of the original host cell (13–16), thereby leaving the niche intact, other studies have suggested that *Leishmania* parasites induce cell death of the infected host cell, thereby increasing the possibility of cell-to-cell transmission (17, 18). In addition, we recently showed that *L*. *major*–infected murine macrophages exhibited signs of cell death (membrane blebbing) shortly before the parasites associated with them were taken up by a new phagocyte (6), suggesting that the infection of new host cells mainly occurs via cell-to-cell transmission from dying phagocytes in vitro. Also, in a human in vitro infection model, we demonstrated that *L*. *major* promastigotes, hiding inside apoptotic neutrophils, can transfer to macrophages, using the neutrophils as Trojan horses (19). However, the findings regarding original host cell death and *Leishmania* cell-to-cell transfer are so far inconclusive and did not investigate the link between infection and cell death on a cellular level, but have focused on the connection between infection-related inflammation and cell death. Therefore, we aimed to investigate host cell death in relation to *L*. *major* exit from and uptake into monocyte-derived phagocytes during ongoing infection in vivo.

Using intravital 2-photon microscopy of the infected skin, we show here an increased apoptosis rate in *L*. *major*–infected phagocytes in the ongoing infection, and direct cell-to-cell transfer to newly recruited phagocytes. This transfer involved the uptake of cell material from the original infected host cell both in vivo and in isolated human cells. In addition, our findings indicated that *L*. *major* proliferation specifically modulates host cell metabolism and drives cell death, thereby enabling the efficient dissemination of the pathogen to new phagocytes.

## Results

### Quantification of L. major uptake by newly recruited monocytes in vivo reveals direct cell-to-cell transfer.

**Figure 1:**
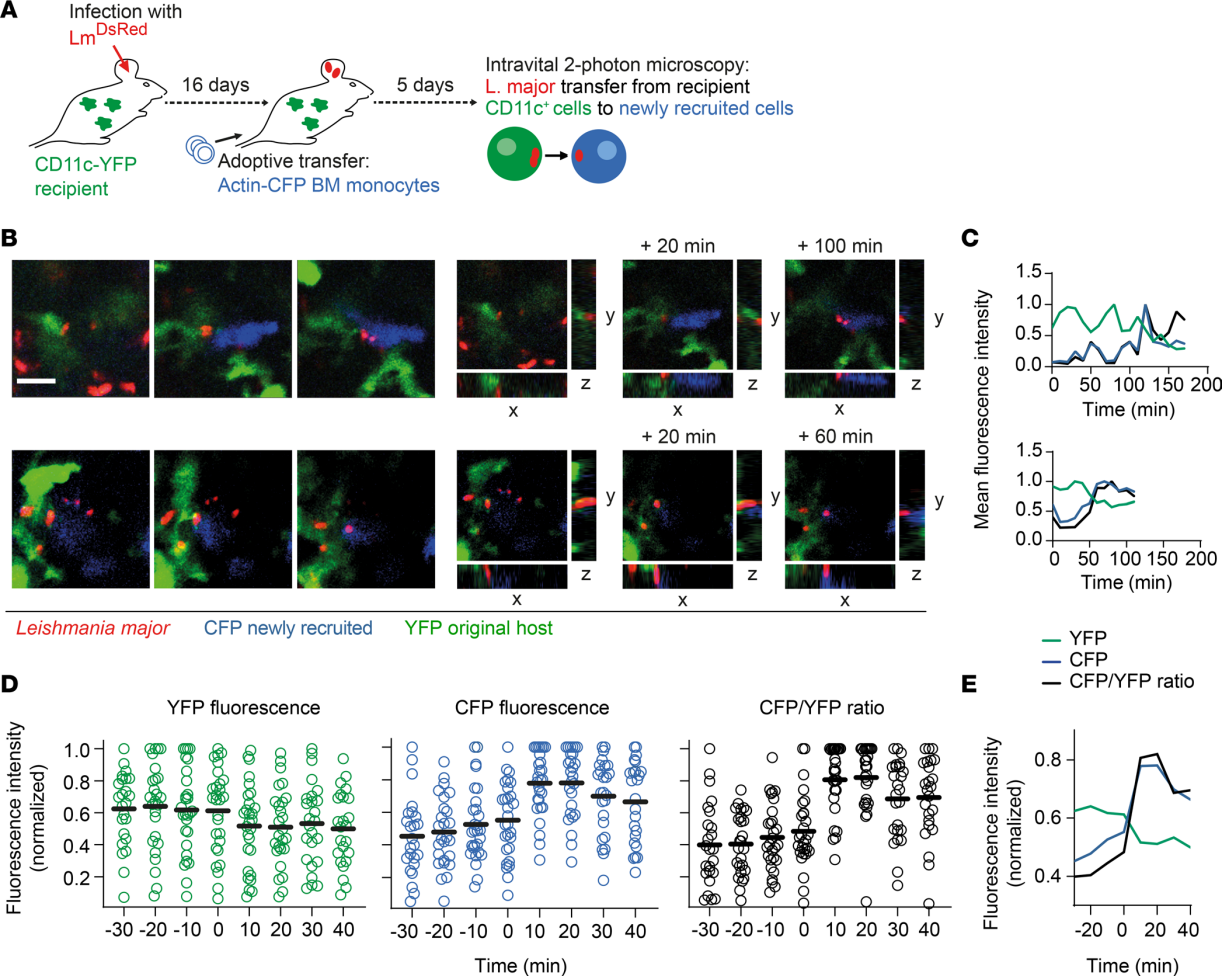
*L*. *major* cell-to-cell transfer is direct in vivo. (**A**) Experimental strategy to determine *Lm^DsRed^* transfer from recipient CD11c-YPF cells into newly recruited actin-CFP bone marrow (BM) cells by intravital 2-photon microscopy in vivo. (**B** and **C**) *Z*-projections (left) and single *xy* image planes with *xz*/*yz* reconstructions (right) showing 2 examples (**B**) with quantification (**C**) of an *L*. *major* cell-to-cell transfer event in the ear dermis of an anesthetized mouse. Projections consist of 8 and 5 slices, respectively, of 3-μm-spaced *Z*-stacks taken longitudinally every 10 minutes. Scale bar: 15 μm. (**D**) Fluorescence intensity of YFP (green), CFP (blue), and CFP/YFP ratio (black) around parasites undergoing cell-to-cell transfer over time of all transfer events obtained from 6 animals imaged. MFI normalized to the minimum and maximum of each parasite track is shown. Each dot represents 1 time point of a transfer event. Horizontal bars represent the mean. (**E**) Overlay of mean values data shown in **D**. Data pooled from 2 independent experiments.

We had recently found evidence that *L*. *major* parasites can transfer directly from one host cell to the next (6). To further investigate and quantify *L*. *major* cell transmission in vivo, we infected CD11c-YFP reporter mice with 2 × 10^5^ DsRed-expressing *L*. *major* (*Lm^DsRed^*) in the ear dermis for 16 days, then adoptively transferred bone marrow cells from constitutively CFP-expressing actin-CFP mice, and subjected the mice to intravital 2-photon imaging after 5 days (Figure 1A). We could readily observe the transfer of DsRed-expressing parasites from CD11c-YFP–expressing recipient cells into newly recruited CFP-expressing cells (Figure 1B). When quantifying the YFP and CFP fluorescence surrounding a parasite during cell-to-cell transfer over time, we observed an immediate drop in YFP fluorescence concomitantly with CFP fluorescence increase, leading to an immediate increase in the CFP/YFP ratio over time (Figure 1C). Quantification of all transition events detected revealed that YFP fluorescence around the parasite did not significantly change before the uptake by CFP-expressing cells but only at the time point of the most pronounced increase in CFP fluorescence (Figure 1, D and E). These observations suggested that the transfer of *L*. *major* parasites from one host cell to the next is direct, with no extracellular phase detectable in vivo.

### Original host cell material is taken up by newly infected cells.

**Figure 2:**
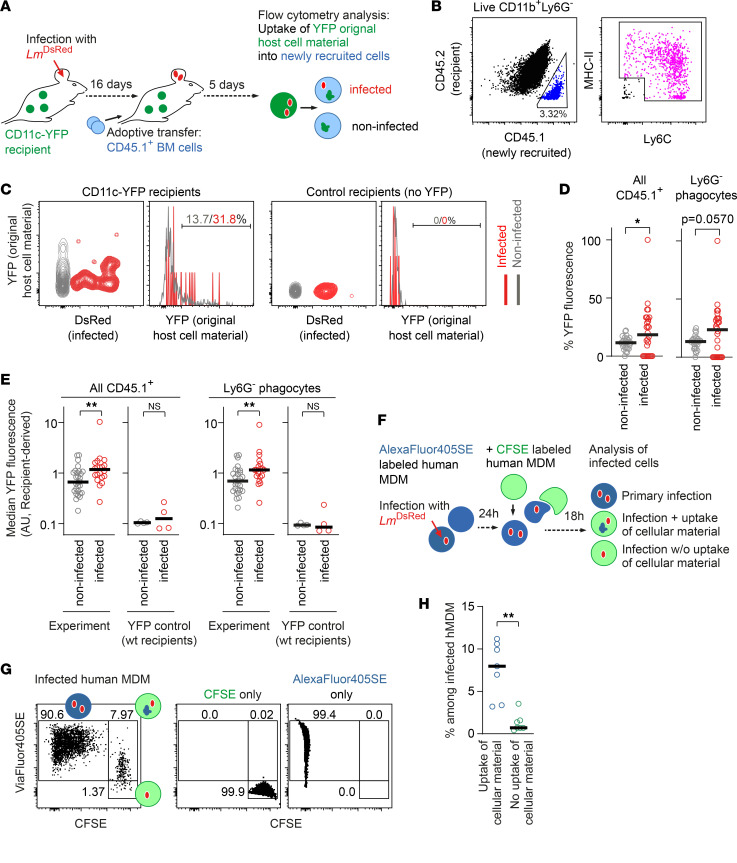
*L*. *major*–infected newly recruited cells take up more YFP^+^ original host cell material compared with uninfected cells. (**A**) Experimental strategy to determine YFP^+^ material uptake into newly recruited *Lm^DsRed^*-infected and uninfected bone marrow cells in vivo. (**B**) Gating on CD45.1^+^ (newly recruited, blue) and CD45.2^+^ (recipient, black) cells and Ly6G^–^ phagocytes. (**C**) Gating on median YFP fluorescence in newly recruited CD45.1^+^ infected (red) and uninfected (gray) cells for CD11c-YFP (left panel) and control (right panel) recipient mice. (**D** and **E**) Quantification of percentage of YFP fluorescence (**D**) and median YFP fluorescence (**E**) in all CD45.1^+^ cells (left panels) and Ly6G^–^ phagocytes (right panels) within live CD11b^+^Ly6G^–^ newly recruited bone marrow cells for CD11c-YFP and control recipient mice (**E** only). Each dot represents 1 mouse ear. Horizontal bars denote the median. Data pooled from 3 independent experiments. (**F**) Experimental strategy to determine Alexa Fluor 405-SE^+^ material uptake from primary *Lm^DsRed^*-infected hMDMs into newly infected CFSE^+^ hMDMs in vitro. (**G**) Gating on primary infected (Alexa Fluor 405-SE^+^ CFSE^–^), newly infected without cellular material uptake (ViaFluor 405 SE^–^ CFSE^+^), and newly infected with cellular material uptake (Alexa Fluor 405-SE^+^ CFSE^+^) hMDMs. Uninfected cells stained with either CFSE or Alexa Fluor 405-SE are shown as controls. (**H**) Quantification of percentage of infected hMDMs without cellular material uptake and with cellular material uptake. Each dot represents 1 sample. Horizontal bars denote the median. *P < 0.05, **P < 0.01 by paired *t* test.

We next sought to analyze possible mechanisms of cell-to-cell transfer in vivo. We and others had found evidence that *L*. *major* transfer to new host cells might occur from dying phagocytes. In vivo, this has only been shown for infected neutrophils at the very early phase of the infection (5, 6, 18). Therefore, we examined whether the uptake of original host cell material is accompanied by the uptake of the parasite into recruited monocytes. To this end, we infected CD45.2^+^ CD11c-YFP reporter mice with 2 × 10^6^ DsRed-expressing *L*. *major* in the ear dermis for 16 days, and then adoptively transferred bone marrow cells from CD45.1^+^ wild-type mice. Five days after transfer, cells isolated from the infected ears were analyzed by flow cytometry (Figure 2A and Supplemental Figure 1A; supplemental material available online with this article; https://doi.org/10.1172/jci.insight.169020DS1). Newly recruited monocytes could be identified according to their CD45.1 expression (Figure 2B), and exhibited an increase in YFP fluorescence in CD45.2 CD11c-YFP recipient mice as compared with those transferred into CD45.2 nonfluorescent recipients, suggesting they had taken up YFP at the site of infection (Figure 2C). Interestingly, we observed significantly more YFP fluorescence in infected compared with uninfected CD45.1^+^ cells (Figure 2, D and E). To test for human relevance, we studied the transfer of cellular material using an in vitro coincubation assay with human monocyte-derived macrophages (hMDMs). To this end, Alexa Fluor 405-SE–labeled *Lm^DsRed^*-infected hMDMs were coincubated with CFSE-labeled uninfected hMDMs for 18 hours, and the transfer of cellular material into CFSE-labeled uninfected hMDMs was analyzed by flow cytometry analysis (Figure 2F and Supplemental Figure 1B). Infected cells could be clearly distinguished into cells that were primary-infected (Alexa Fluor 405-SE^+^ CFSE^–^), newly infected without uptake of cellular material (Alexa Fluor 405-SE^–^ CFSE^+^), and newly infected with uptake of cellular material (Alexa Fluor 405-SE^+^ CFSE^+^) (Figure 2G). In line with our in vivo data, the percentage of infected hMDMs was significantly higher for infected cells containing cellular material of the primary infected host compared with infected cells containing no cellular material of the primary infected host (Figure 2H). These results indicate that uptake of the parasite by newly recruited monocytes is occurring concomitantly with uptake of material from the originally infected host cell.

### An in vivo cell death reporter system shows higher apoptosis in infected than in uninfected phagocytes at the site of infection.

**Figure 3:**
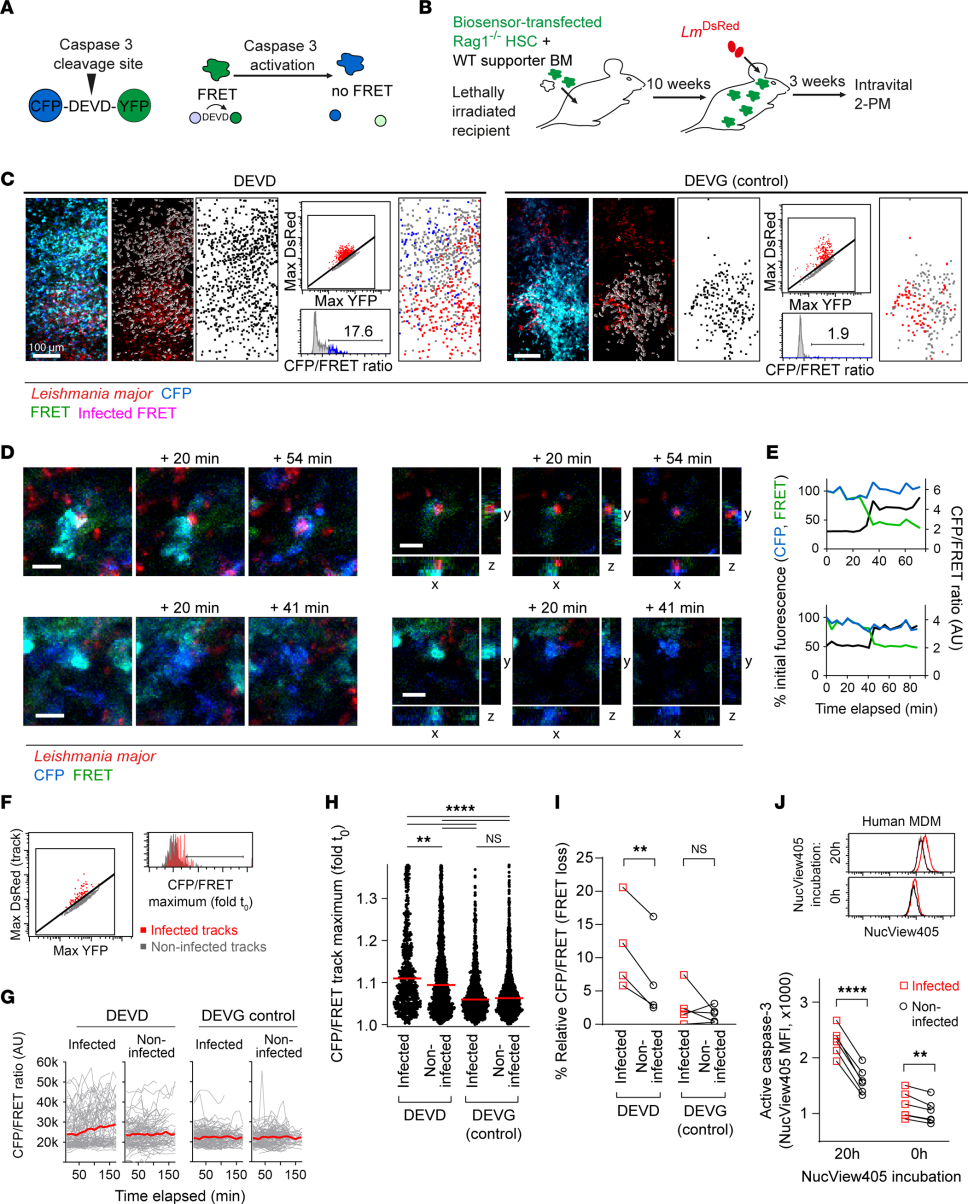
More apoptosis in *L*. *major*–infected cells. (**A**) In vivo quantification of cell death dynamics. DEVD, caspase-3 recognition site. (**B**) *Rag1*^–/–^ hematopoietic stem cells (HSC) transduced with caspase-3 reporter DEVD or noncleavable control (DEVG) were FACS-sorted and transferred into lethally irradiated hosts. (**C**) *Z*-projections of infected ear dermis of CFP-DEVD-YFP (left) and CFP-DEVG-YFP control (right) mice. Eighteen slices of 3-μm-spaced *Z*-stacks are projected. Scale bars: 100 μm. Gray, 3D surface plot of detected reporter-expressing cells; black, mapped cell positions; red, infected cells; blue, cells with increased CFP/FRET ratio; pink, infected cells exhibiting FRET loss (map only). (**D**) *Z*-projections (left) and *xy*/*xz*/*yz* sections (right) of *Lm^DsRed^*-infected (top panels) and uninfected (bottom panels) apoptotic cells over time. Eleven and 14 slices (3-μm-spaced *Z*-stacks) taken longitudinally are projected. Scale bars: 10 μm. (**E**) Percentage of initial CFP and FRET fluorescence and normalized CFP/FRET ratio over time of cells in **D**. (**F**) Gating of cell tracks infected (left) or losing FRET (right), in infected (red) and uninfected (gray) cells. (**G**) FRET loss in infected (left panels) and uninfected (right panels) cells for CFP-DEVD-YFP (left) and CFP-DEVG-YFP control (right) mice. Fifty randomly selected tracks of at least 200 tracks analyzed per condition are shown. (**H**) FRET loss in infected and uninfected CFP-DEVD-YFP and CFP-DEVG-YFP control mice. Each symbol represents 1 track. Horizontal bars denote the median. (**I**) FRET loss in infected and uninfected cell populations from 4–5 animals imaged. Each symbol pair represents 1 mouse ear. (**J**) NucView405 (caspase-3 activity) in *Lm^DsRed^*-infected (red) and uninfected (black) hMDMs 0 hours and 20 hours after incubation. Each symbol pair represents 1 donor. **P < 0.01, ****P < 0.0001 by Kruskal-Wallis with Dunn’s posttest in **H**, and paired 2-way ANOVA with Bonferroni’s posttest in **I** and **J**.

To further investigate the fate of host cells in the context of parasite infection, we aimed to determine the dynamics of programmed cell death induction during intravital 2-photon microscopy of the *L*. *major* infection site in the ear dermis of the mouse. For this, we used a genetically encoded reporter system in inflammatory cells recruited to the skin by retrovirally transducing *Rag1^–/–^* hematopoietic stem cells (HSCs) with a CFP-DEVD-YFP construct (20, 21). This construct is sensitive to cleavage by caspase-3 at the specific Asp-Glu-Val-Asp site linking a Förster resonance energy transfer (FRET) donor (CFP) and acceptor (YFP). Upon the induction of apoptosis, active caspase-3 cleaves the donor from the acceptor, resulting in an increased CFP/FRET ratio (Figure 3A). A noncleavable CFP-DEVG-YFP control construct (22) was used to determine specific caspase-3–dependent cleavage. The transfected *Rag1^–/–^* HSCs together with wild-type supporter bone marrow were used to reconstitute lethally irradiated recipient mice, resulting in animals that expressed the reporter constructs specifically in the non-lymphocyte immune cell compartment. These mice were infected with 2 × 10^5^ DsRed-expressing *L*. *major* and imaged 3 weeks later using intravital 2-photon microscopy (Figure 3B). In the blood, reporter-expressing cells exhibited a small fraction of cells with FRET loss in the CFP-DEVD-YFP bone marrow chimeric mice, but not in the bone marrow chimeric mice containing the CFP-DEVG-YFP control construct (Supplemental Figure 2, A and B). As expected, time-lapse imaging using 2-photon microscopy at the site of infection showed an accumulation of reporter-expressing bone marrow–derived cells. Automated segmentation and tracking of these cells over time enabled us to map, identify, and localize infected and FRET-losing cells by converting the extracted imaging data to cytometry data sets using *DISCit* software (23). This revealed FRET loss in reporter-expressing cells for the CFP-DEVD-YFP, but not the CFP-DEVG-YFP controls (Figure 3C). Also, we could localize individual cells undergoing FRET loss over time (Figure 3, D and E). Strikingly, when we compared *L*. *major*–infected with uninfected cells (Figure 3F), we found progressive FRET loss in the infected cell population, which was higher in comparison with the uninfected cell population (Figure 3, G and H). Again, no FRET loss was observed in the control CFP-DEVG-YFP bone marrow chimeras (Figure 3, H and I). Thus, these data indicate that *L*. *major*–infected cells undergo more apoptosis as compared with uninfected cells. To support these findings, we made use of a caspase-3 reporter assay to measure caspase-3 activity in *Lm^DsRed^*-infected hMDMs by flow cytometry. Indeed, we observed significantly higher NucView405 mean fluorescence, indicating caspase-3, in infected compared with uninfected hMDMs. Strikingly, this was already observed right after staining with the caspase-3 reporter, but was even more apparent 20 hours after incubation with the reporter (Figure 3J and Supplemental Figure 2C). Therefore, our data indicated that *L*. *major* infection is related to caspase-3 activity and thereby to apoptosis, both in vivo and in vitro.

### L. major–infected cells containing high-proliferating pathogens undergo apoptosis and drive cell-to-cell transfer.

**Figure 4:**
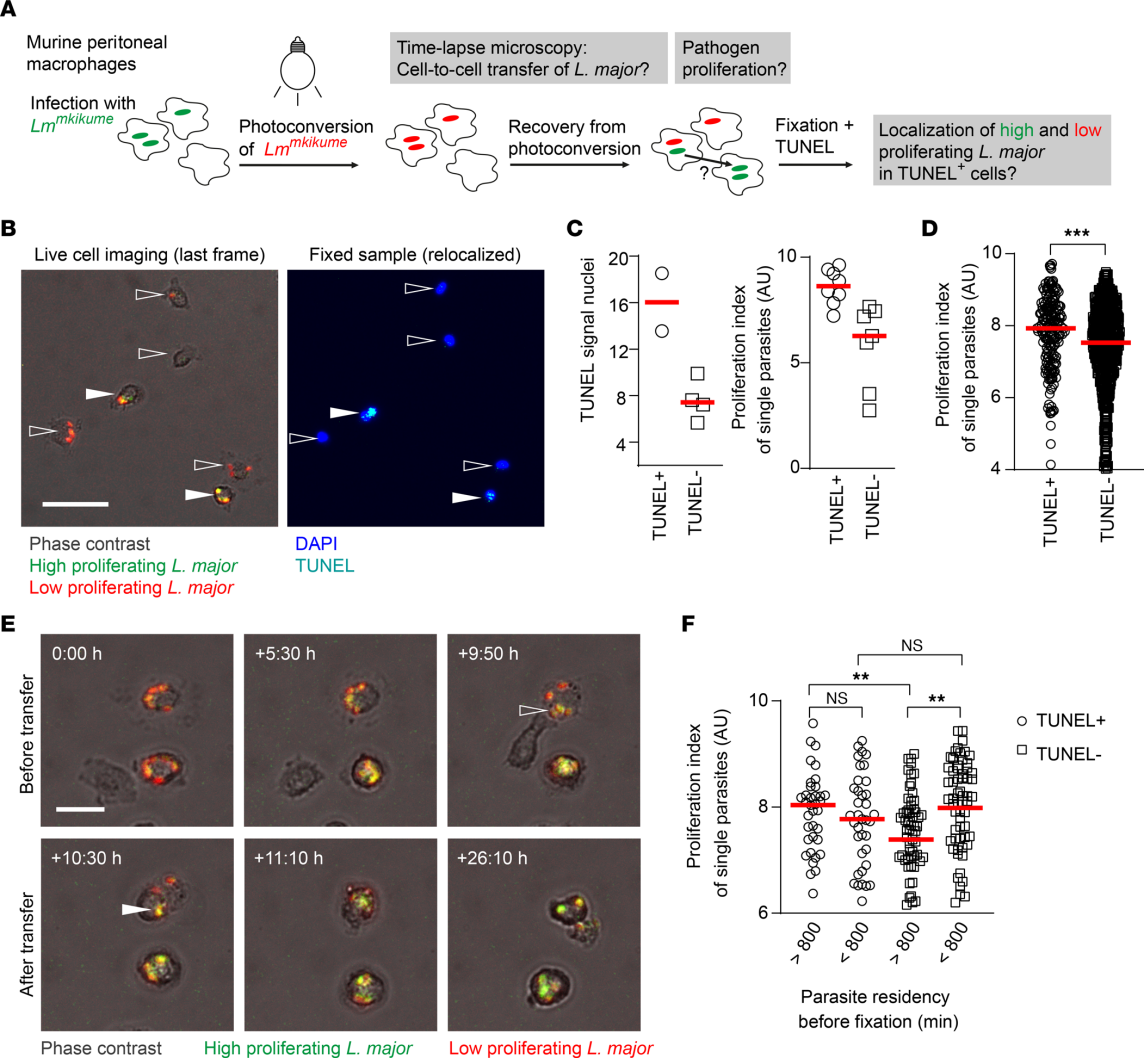
*L*. *major* proliferation rate affects the fate of the infected host cell. (**A**) Experimental setup for in vitro analysis of parasite proliferation and host cell death using a photoconversion-based pathogen proliferation biosensor (*Lm^mKikume^*) and wide-field imaging. (**B**) One example of high-proliferating (green) *Lm^mKikume^* parasites colocalizing with TUNEL^+^ murine intraperitoneal (IP) macrophages. One frame at the end of live cell imaging (left) and TUNEL staining after fixation (right) of the same site are shown. TUNEL^+^ cells are marked with filled arrows, and TUNEL^–^ cells are marked with open arrows. Scale bar: 50 μm. (**C**) Quantification of TUNEL signal (left) and proliferation index (right) within the IP macrophages shown in **B**. Each symbol represents 1 individual cell (left) or parasite (right). Horizontal bars denote the median. (**D**) Quantification of proliferation index in all TUNEL^+^ and TUNEL^–^ murine IP macrophages analyzed. Each symbol represents 1 individual parasite. Data were pooled from 2 independent microscopy experiments. Horizontal bars denote the median. (**E**) One example of an *Lm^mKikume^* cell-to-cell transfer event between 2 murine IP macrophages. The transferring *Lm^mKikume^* parasites are indicated with an open arrow in the donor host macrophage and with a filled arrow in the recipient macrophage. Scale bar: 20 μm. (**F**) Quantification of proliferation index in TUNEL^+^ and TUNEL^–^ murine IP macrophages distinguished based on long-term (>800 minutes before fixation) and recent (<800 minutes before fixation) parasite residency. Note that long-term intracellular residency in TUNEL^–^ cells involves low parasite proliferation. Each symbol shows 1 individual parasite. Data were pooled from 2 independent microscopy experiments. Horizontal bars denote the mean. **P < 0.01, ***P < 0.001 by Mann-Whitney test.

Our previous findings had suggested that high-proliferating *L*. *major* parasites undergo more cell-to-cell transmission than low-proliferating parasites (6). Since we had observed enhanced apoptosis in infected cells, we next aimed to investigate *L*. *major* proliferation rate in relation to cell death of the infected host cells in vitro. To do so, we infected murine intraperitoneal macrophages with mKikume-expressing *Lm*^SWITCH^ parasites, which express a photoconversion-based proliferation biosensor (24). In brief, the parasites in the infected macrophages were photoconverted from green to red and imaged by wide-field microscopy for 48 hours. This enabled us to track cell-to-cell transfer of the parasites, and to determine pathogen proliferation as a function of recovery from photoconversion (6). Furthermore, the use of gridded microscopy dishes allowed us to fix the cells and identify apoptotic macrophages by TUNEL staining following live cell imaging (Figure 4A). We detected higher *L*. *major* proliferation in TUNEL^+^ compared with TUNEL^–^ macrophages (Figure 4, B and C), suggesting that high parasite proliferation is correlated to cell death of the original host cells (Figure 4, B–D). Time-resolved analysis of cell-to-cell transfer events prior to fixation (Figure 4E) revealed that parasite proliferation was high not only in TUNEL^+^ cells, but also in TUNEL^–^ cells that had been infected only recently (<800 minutes; Figure 4F). In contrast, TUNEL^–^ cells found infected for longer periods (>800 minutes) harbored parasites with significantly lower proliferation. As shown previously in vivo (6), we did not detect any correlation between pathogen burden and intracellular pathogen proliferation rate (Supplemental Figure 3, A and B), indicating that the differences of intracellular residence time before apoptosis were attributable to pathogen proliferation and not pathogen burden. Taken together, these data suggested that long-term residency of *L*. *major* parasites within macrophages was related to low parasite proliferation.

### Proliferation-competent pathogens induce cell death in infected macrophages.

**Figure 5:**
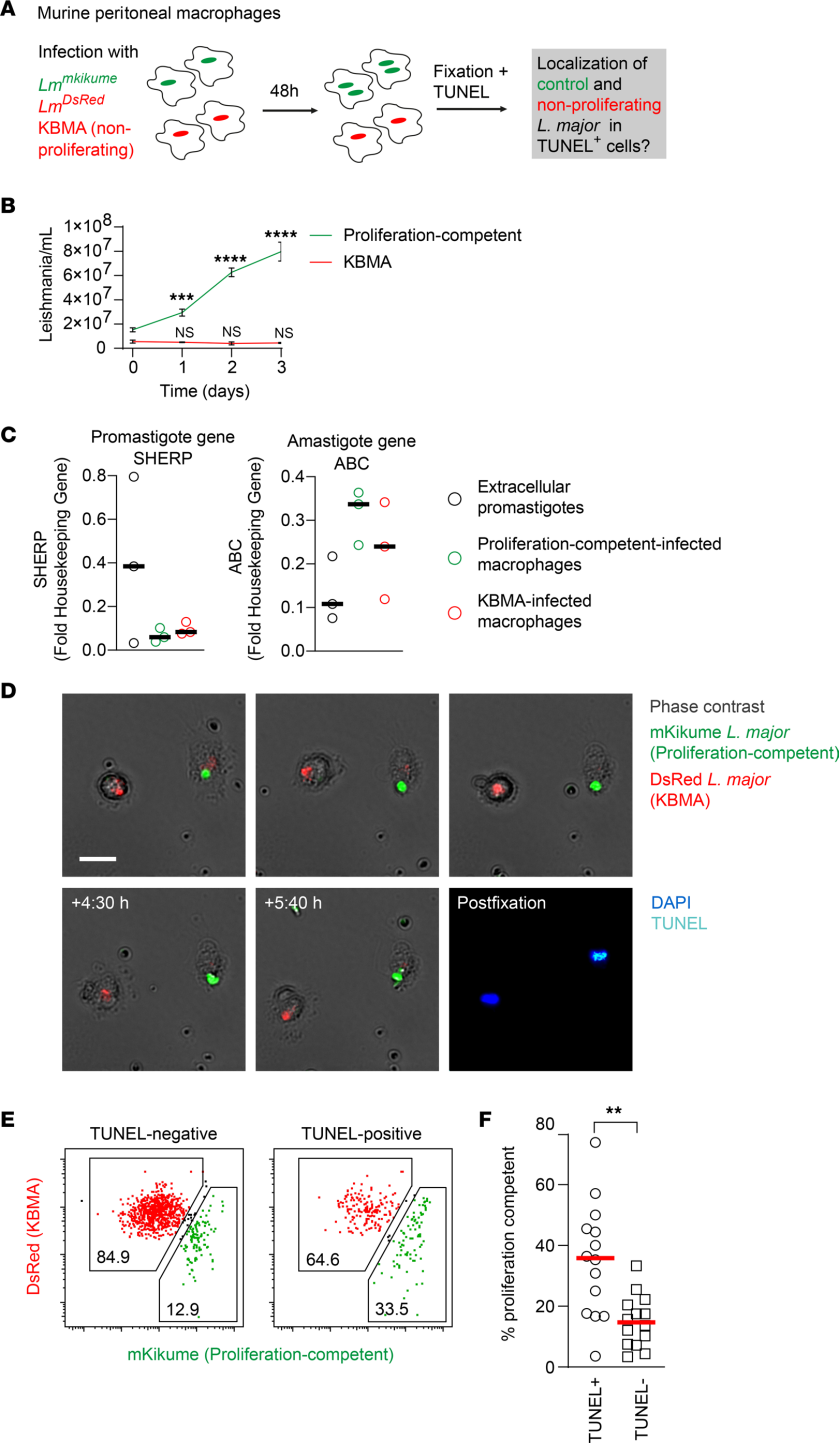
Proliferation-competent *L*. *major* parasites induce cell death in infected macrophages. (**A**) Experimental setup for in vitro analysis of host cell death using *Lm^mKikumeGreen^* proliferation-competent and *Lm^DsRed^* KBMA parasites and wide-field imaging. (**B**) Growth curve of proliferation-competent (green) and KBMA proliferation-incompetent (red) parasites. (**C**) Promastigote (*SHERP*) and amastigote (*ABC*) gene expression (normalized to *NMT* as a housekeeping gene) in extracellular promastigotes and proliferation-competent and KBMA proliferation-incompetent parasites as measured by qPCR. Each symbol represents 1 sample. Data represent 3 independent samples for each condition. Horizontal bars denote the median. (**D**) Selected frames from live cell imaging and TUNEL staining after fixation of the same site are shown, revealing TUNEL^+^ (cyan) murine IP macrophages colocalizing with proliferation-competent (green) but not KBMA proliferation-incompetent (red) parasite. Scale bar: 20 μm. (**E**) Selection of *Lm^mKikumeGreen^* proliferation-competent and *Lm^DsRed^* KBMA parasites in TUNEL^–^ (left) and TUNEL^+^ (right) cells according to green and red parasite fluorescence. All measured infected cells are shown individually. (**F**) Quantification of percentage of proliferation-competent parasites in all TUNEL^+^ and TUNEL^–^ murine IP macrophages. Each symbol shows 1 field of view imaged over time and relocalized afterward for TUNEL staining, with at least 3 TUNEL^+^ and at least 6 TUNEL^–^ cells per imaged field analyzed according to the criteria shown in **E**. Horizontal bars denote the mean. **P < 0.01, ***P < 0.001, ****P < 0.0001 by paired *t* test.

The observation that high-proliferating *L*. *major* parasites were found mainly in apoptotic or newly infected cells raised the question of whether pathogen proliferation might induce cell death in infected macrophages or is a result of cell death. In order to investigate whether a predefined parasite proliferation affects the fate of the infected host in a direct manner, we aimed to modify parasite proliferation for infection of murine intraperitoneal macrophages and murine bone marrow–derived macrophages (BMDMs). To do so, we made use of killed but metabolically active (KBMA) *L*. *major* parasites (25–27). These parasites are not able to proliferate due to low-grade DNA cross-linking, but largely retained their fluorescence (Figure 5, A and B, and Supplemental Figure 3, C and D) and could be shown by quantitative PCR to switch to amastigote-specific gene expression upon infection of macrophages (Figure 5C). To study the fate of KBMA *L*. *major*–infected macrophages versus macrophages infected with proliferation-competent parasites side by side, KBMA parasites labeled by DsRed expression and untreated, mKikume green–expressing parasites were used to coinfect murine intraperitoneal macrophages. The infected macrophages were imaged by wide-field microscopy for 48 hours using gridded microscopy dishes to identify apoptotic macrophages by TUNEL staining after the live cell imaging (Figure 5D). We observed cell death both by TUNEL staining and by visual signs of cell death (membrane blebbing; Figure 5D). Significantly more proliferation-competent, mKikume green–expressing parasites were observed in the TUNEL^+^ compared with the TUNEL^–^ macrophages, suggesting that *L*. *major* proliferation induces cell death of the infected host (Figure 5, E and F).

### L. major intracellular proliferation rate modifies host cell metabolic pathways.

**Figure 6:**
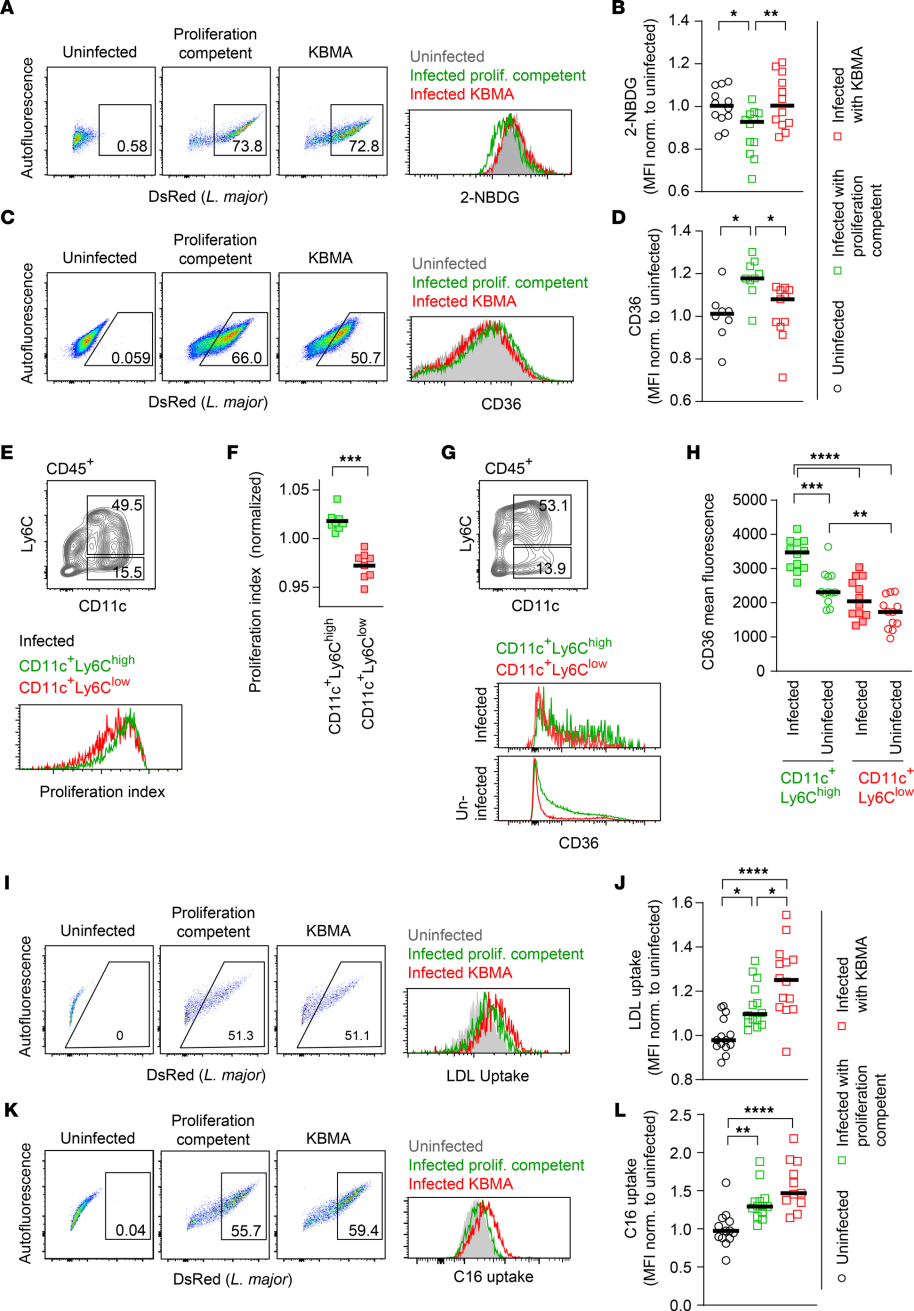
*L*. *major* intracellular proliferation rate modifies host cell metabolic pathways. (**A**–**D**) Gating and normalized fluorescence for 2-NBDG uptake (**A** and **B**) and CD36 (**C** and **D**) in uninfected (gray), *Lm^DsRed^* proliferation-competent–infected (green), and *Lm^DsRed^* KBMA–infected (red) BMDMs. Each symbol shows 1 individual biological replicate. Horizontal bars denote the mean. Data pooled from 3 independent experiments. *P < 0.05, **P < 0.01 by 1-way ANOVA with Bonferroni’s posttest. (**E**) Gating for CD11c^+^Ly6C^hi^ and CD11c^+^Ly6C^lo^ CD45.1^+^ monocytes (top panel) and histogram plots showing proliferation index in infected CD11c^+^Ly6C^hi^ (green) and CD11c^+^Ly6C^lo^ (red) (bottom panel) CD45.1^+^ cells. (**F**) Quantification of proliferation index normalized to the total proliferation index in each sample (see Methods) in infected CD11c^+^Ly6C^hi^ (green) and CD11c^+^Ly6C^lo^ (red) monocytes in newly recruited CD45.1^+^ cells analyzed according to gating shown in **E**. Each symbol represents 1 mouse ear. Horizontal bars denote the mean. Data pooled from 2 independent experiments. ***P < 0.001 by paired *t* test. (**G**) Gating for CD11c^+^Ly6C^hi^ and CD11c^+^Ly6C^lo^ monocytes (top panel) and histogram plots showing CD36 expression in infected and uninfected CD11c^+^Ly6C^hi^ (green) and CD11c^+^Ly6C^lo^ (red) (bottom panel) CD45^+^ cells. (**H**) MFI for CD36 expression in infected (squares) and uninfected (circles) CD11c^+^Ly6C^hi^ (green) and CD11c^+^Ly6C^lo^ (red) CD45^+^ monocytes analyzed according to gating shown in **G**. Each symbol represents 1 mouse ear. Horizontal bars denote the mean. Data pooled from 2 independent experiments. **P < 0.01, ***P < 0.001, ****P < 0.0001 by 1-way ANOVA with Bonferroni’s posttest. (**I**–**L**) Gating and normalized LDL (**I** and **J**) and long-chain fatty acid (C16) (**K** and **L**) uptake in uninfected (gray), *Lm^DsRed^* proliferation-competent–infected (green), and *Lm^DsRed^* KBMA–infected (red) BMDMs. Each symbol shows 1 individual sample. Data pooled from 3 independent experiments. Horizontal bars denote the mean. *P < 0.05, **P < 0.01, ****P < 0.0001 by 1-way ANOVA with Bonferroni’s posttest.

In order to analyze host cellular changes related to infection with proliferation-competent versus KBMA parasite, we set up a flow cytometry–based analysis approach. Murine BMDMs were used for this purpose in order to yield sufficient numbers of host cells for infection and analysis. Annexin V staining (28) 48 hours after infection revealed comparable cell death between uninfected and KBMA or proliferation-competent parasite–infected host cells, probably due to lower adherence of dying cells in the course of the experiment (Supplemental Figure 4). However, this approach enabled us to measure the impact of *L*. *major* proliferation capacity on host cellular metabolism on a single-cell level. To this end, we studied glucose uptake and transport by the fluorescent tracer 2-(*N*-(7-nitrobenz-2-oxa-1,3-diazol-4-yl)amino)-2-deoxyglucose (2-NBDG) and expression of glucose transporter 1 (GLUT1). The macrophages infected with either KBMA *L*. *major* or proliferation-competent parasites were compared with uninfected cells by flow cytometry 48 hours after infection (Figure 6A and Supplemental Figure 5, A–C). While no significant differences were observed in 2-NBDG uptake between uninfected and KBMA-infected macrophages, macrophages infected with proliferation-competent *L*. *major* exhibited significantly less 2-NBDG uptake (Figure 6, A and B). This suggested that infection with proliferating parasites decreases glucose uptake in the host cell. However, no significant differences were observed in GLUT1 expression between any of the conditions (Supplemental Figure 5, B and C), suggesting that the changes in glucose uptake induced by proliferation-competent pathogens are not achieved via marked changes in surface expression of the transporter GLUT1.

In addition to glucose uptake, we analyzed the expression of CD36, a scavenger receptor involved in high-affinity tissue uptake of long-chain fatty acids (29, 30). We found that expression of CD36 was significantly increased in macrophages infected with proliferation-competent parasites as compared with both uninfected and KBMA-infected cells (Figure 6, C and D), suggesting that pathogen proliferation might increase CD36 expression. To validate these findings in vivo, we analyzed CD11c^+^Ly6C^hi^ and CD11c^+^Ly6C^lo^ monocytes isolated from the site of infection with *Lm*^SWITCH^. When we determined pathogen proliferation in vivo (6), CD11c^+^Ly6C^hi^ monocytes exhibited higher intracellular pathogen proliferation than CD11c^+^Ly6C^lo^ monocytes (Figure 6, E and F, and Supplemental Figure 5D). Notably, CD36 expression in cells isolated from the infection site was not only generally higher in CD11c^+^Ly6C^hi^ monocytes, but also exhibited a substantial increase in infected compared with uninfected CD11c^+^Ly6C^hi^ monocytes. This was, in contrast, not the case for CD11c^+^Ly6C^lo^ monocytes (Figure 6, G and H, and Supplemental Figure 5, E and F). This suggested that, also in vivo, infection with high-proliferating pathogen correlated with higher CD36 expression. In order to evaluate whether differential pathogen proliferation states resulted in distinct lipid uptake behaviors in infected cells, we analyzed the uptake of low-density lipoprotein (LDL) and long-chain (C16) and short-/medium-chain (C1/12) fatty acids into uninfected as well as KBMA and proliferation-competent *L*. *major*–infected murine BMDMs. Both LDL (Figure 6, I and J) and long-chain (Figure 6, K and L), but not short-chain, fatty acid uptake (Supplemental Figure 5, G and H) was increased upon *L*. *major* infection. Strikingly, KBMA-infected monocytes showed a slightly higher uptake of LDL and long-chain fatty acids as compared with cells infected with proliferation-competent *L*. *major*. This suggested that the increased CD36 expression might be compensatory as a result of different lipid uptake rates linked with pathogen proliferation (31), and underlined that *L*. *major* proliferation differentially modulated metabolic pathways in the infected host phagocytes.

Together, our data suggest that pathogen proliferation is linked with cell-to-cell transfer via manipulation of the host cell resulting in metabolic changes and an increased probability of cell death.

## Discussion

The abilities of *L*. *major* parasites to proliferate within and to spread among phagocytes are the main survival strategies of the parasite, but have hardly been studied during ongoing infection. A possible mechanism involved in *L*. *major* cell-to-cell transfer is cell death of the infected host cell. However, findings regarding cell death during *Leishmania* infection are inconsistent, but elucidating these processes might identify novel targets for future treatment approaches. Therefore, we aimed to investigate original host cell death in relation to *L*. *major* exit from and uptake into monocyte-derived phagocytes during ongoing infection in vivo. In this study, we show that *L*. *major* infection and intracellular proliferation increase the probability of host cell death in vivo, and thus promote pathogen exit and cell-to-cell transfer.

Our data suggest that transfer of *L*. *major* parasites from one host cell to the next is direct, with no extracellular phase detectable in vivo. In line with this, direct uptake from infected host cells into new cells in vitro (6) and *L*. *amazonensis* amastigote transfer from cell to cell without full exposure to the extracellular milieu were reported (18). Also, we observed uptake of *L*. *major* parasites by newly recruited monocytes together with transfer of original host cell material. Interestingly, our previous findings suggest that the original host cell is phagocytosed by the newly infected host cell upon parasite transfer in vitro. While the uptake of neutrophil material together with parasites by recruited monocyte-derived macrophages and dendritic cells has been demonstrated in a variety of studies (32–35), the transit between infected monocyte-derived cells in an established infection had been less well elucidated. Our data now show that also the transfer from infected CD11c-expressing cells, most probably inflammatory monocytes (6), involves the uptake of original host cell material. Importantly, we could validate our findings from mice in an in vitro system of human monocytes, illustrating that in both preclinical in vivo and human in vitro systems, uptake of original host cell material is involved in the cell-to-cell transfer of the parasite.

By both live cell imaging and intravital 2-photon imaging in the living tissue, we observed more apoptosis in *L*. *major*–infected cells as compared with uninfected cells. This is in contrast to a number of previous studies that suggested that *Leishmania* inhibits apoptosis. For example, Roy and colleagues showed that *L*. *donovani* infection inhibits the programmed cell death-1 receptor (13). Other studies suggested that *L*. *donovani* activates the antiapoptotic AKT signaling pathway (14, 36, 37) or the antiapoptotic protein myeloid cell leukemia 1 (15) or increases Bcl-2 in macrophages (16). However, these studies investigated mainly the prevention of induced apoptosis in vitro, or cell death irrespective of the cellular infection status. In line with our findings of the present study, there are also a number of reports showing that *Leishmania* induces apoptosis. DaMata and colleagues demonstrated that *L*. *amazonensis* induces phosphatidylserine exposure, DNA cleavage into nucleosomal size fragments, and consequent hypodiploidy, all indicating apoptosis. In addition, the same study showed that *L*. *amazonensis*–induced macrophage apoptosis was associated with activation of caspases-3, -8, and -9 (17). Furthermore, we recently showed that *L*. *major*–infected murine macrophages exhibited signs of cell death (membrane blebbing) shortly before the parasites associated with them were taken up by a new phagocyte, suggesting that the infection of new host cells mainly occurs via cell-to-cell transmission from dying phagocytes in vitro (6). Moreover, multidimensional live imaging of long-term-infected macrophages demonstrated that *L*. *amazonensis* amastigotes underwent cell-to-cell transfer when the original host macrophage showed signs of imminent apoptosis in vitro (18).

The observation of higher parasite proliferation in dying host cells suggests that *L*. *major* proliferation could result in death of the infected phagocyte. Together with our murine and human data showing more apoptosis in infected compared with uninfected cells, we hypothesized that proliferating *L*. *major* parasites induce cell death of the original host cell, thereby promoting transfer to a new phagocyte. This is in line with earlier data obtained by our group showing that high-proliferating *L*. *major* parasites undergo more cell-to-cell transfer as compared with low-proliferating parasites (6). Also in support of this hypothesis, our data indeed showed that host cells are only able to survive for more than 72 hours after infection if the proliferation rate of the parasite inside the cell is low, suggesting that long-term residency of *L*. *major* parasites within macrophages is related to low parasite proliferation.

Although we used a biosensor specific for caspase-3 in vivo, we are not able to formally exclude an involvement of other regulated cell death pathways, such as necroptosis and pyroptosis, in *L*. *major* infection and cell-to-cell transfer. Further studies are needed to explore the role of these pathways in *L*. *major* spread among phagocytes. In this regard, previous studies have demonstrated that necroptosis, more specifically RIPK1-RIPK3-MLKL–associated necroptosis, is important for neutrophil death during *L*. *infantum* infection (38) and for macrophage death during *L*. *braziliensis*, *L*. *amazonensis*, and *L*. *major* infection (39, 40). In addition, it was shown that the inflammasome was important for restriction of parasite replication during infection with *L*. *amazonensis*, *L*. *braziliensis*, and *L*. *infantum* *chagasi*, but not during *L*. *major* infection (41). Moreover, other studies have suggested that host macrophage pyroptosis may contribute to *Leishmania* dissemination (42) and that *L*. *amazonensis* or *L*. *donovani* infection suppresses macrophage pyroptosis (43–46). These findings suggest that apoptosis may not be the only form of programmed cell death important in *Leishmania* spread among phagocytes.

We also aimed to decipher whether high *Leishmania* proliferation induces or is an effect of cell death of the donor cell. We had shown earlier that inflammatory monocytes are infected by parasites with especially high pathogen proliferation (6), and that enhanced monocyte recruitment can increase pathogen proliferation (47), suggesting that pathogen proliferation might be at least in part dependent on the availability of newly recruited host cells. With the enhanced cell death in, and pathogen exit from, cells infected with high-proliferating *L*. *major*, the phagocyte subtype could possibly be the main determinant of pathogen proliferation capacity and propensity of cell death. Using KBMA *L*. *major*, which are proliferation-incompetent parasites, we were able to investigate this question beyond pure correlation between *L*. *major* proliferation and cell death of the original host cell. As expected, infected host cells displaying signs of cell death were more often associated with proliferation-competent parasites as compared with proliferation-incompetent parasites, suggesting that *L*. *major* proliferation induces cell death of the infected host. While KBMA parasites most likely reflect only partially the phenotype of low-proliferating pathogens, our data clearly show that modulation of proliferation per se can impact on the probability of host cell death. To our knowledge, we are the first to show this direct association between *Leishmania* parasite proliferation and host cell death.

We show that expression of the glucose transporter GLUT1 is not affected by *L*. *major* infection, which contrasts with data showing that GLUT1-mediated glucose metabolism drives a proinflammatory phenotype (48). While it is possible that *L*. *major* inhibits the increase in GLUT1 that is normally associated with inflammation, this does not seem to depend on the proliferation rate of the intracellular parasite. In contrast to GLUT1 surface expression, we observed reduced 2-NBDG expression in host macrophages infected with proliferating parasites as compared with nonproliferating parasites, indicating reduced glucose uptake as a result of intracellular pathogen proliferation. This suggests that *Leishmania* might inhibit the increase in glycolysis that macrophages display during inflammatory activation (49, 50). While we cannot formally exclude that this reduced uptake is due to initial events of cell death, our findings would be in line with earlier findings showing that *Mycobacterium tuberculosis* limits host glycolysis (51), which has been shown to be important in host defense against the intracellular pathogen (52–54). Therefore, our observations likely pertain beyond *Leishmania* to other intracellular pathogens. Last, we demonstrate increased CD36 expression upon *L*. *major* infection depending on the proliferation rate of the intracellular parasite. Being a central regulator of lipid metabolism, CD36 has important functions in the uptake of fatty acids, but also serves as a scavenger receptor (55, 56). Evidence for changes in lipid regulation is particularly interesting in the context of *Leishmania* since infected cells exhibit cholesterol depletion from their plasma membrane, as well as a modulated gene regulation in cholesterol biosynthetic and trafficking pathways in vitro (57, 58). Moreover, CD36 has been found to drive parasitophorous vacuole maturation (59). Notably, we found that the uptake of LDL and long-chain fatty acids, whose transport is mediated by CD36 (29, 60), differed not only between infected and uninfected, but also between KBMA-infected and proliferation-competent *L*. *major–*infected cells. Interestingly, while infection with proliferation-competent *L*. *major* increased CD36 expression as compared with KBMA infection, the effect seemed to be opposite for LDL and long-chain fatty acid uptake. This suggests that *L*. *major* proliferation might impact infected cells in a way that decreases the net lipid uptake, but induces compensatory CD36 expression (31). An alternative explanation for increased CD36 surface expression in macrophages infected with proliferation-competent parasites might be the involvement of this receptor in the phagocytosis of apoptotic cells (61–63). Specifically, the enhanced phagocytosis of transferring proliferating parasite by CD36^hi^ macrophages could result in the higher signal in the population harboring proliferation-competent pathogen. Nevertheless, this would again favor our hypothesis that pathogen proliferation drives cell death of the original host cell, in line with our previous findings that high-proliferating *L*. *major* parasites undergo more cell-to-cell transfer as compared with low-proliferating parasites (6).

Exit strategies of intracellular pathogens are under intense investigation, and a limited set of general strategies seem to be shared across pathogen species, including induced membrane-dependent exit, active host cell lysis, and induction of different forms of cell death (64–66). However, especially for *Leishmania*, data on the significance of any of these strategies for cell-to-cell transfer are scarce (1). Taken together, we show here that *L*. *major* drives host cell death and cell-to-cell transfer among phagocytes. In addition, our findings indicate that increased *L*. *major* proliferation rate might be involved in these processes. To our knowledge, we are the first to show evidence that *L*. *major* stimulates dissemination among phagocytes through parasite proliferation–dependent cell death, which can serve as a starting point for the creation of innovative treatments that can inhibit the establishment of intracellular pathogens at their site of infection.

## Methods

### Parasites, mice, and infections.

All mice were bred and housed under specific pathogen–free conditions in the central animal facility (ZTL) of the Medical Faculty at Otto von Guericke University Magdeburg. Wild-type CD45.1 (B6.SJL-*Ptprc^a^Pepc^b^*/BoyJ), actin-ECFP [B6.129(ICR)-Tg(CAG-ECFP)CK6Nagy/J], *Rag1^–/–^* (B6.129S7-^Rag1tm1Mom^/J), and CD11c-EYFP [B6.Cg-Tg(Itgax-Venus)1Mnz/J] mice were purchased from The Jackson Laboratory, and wild-type C57BL/6J and B6N-TyrcBrdCrCrl (B6 albino wild-type) mice were obtained from Charles River and used at an age of 8–10 weeks for bone marrow recipients and up to 16 weeks for infection and as donors. All mice had been backcrossed for at least 10 generations onto a C57BL/6 background by the commercial suppliers. Age- and sex-matched animals were used as controls. For all murine in vivo and in vitro experiments, *L*. *major* LRC-L137 V121 wild-type or DsRed parasites were previously described (5). Parasites were grown in M199 medium completed with 10% heat-inactivated FCS, 0.1 mM adenine, 1 mg/mL biotin, 5 mg/mL hemin, and 2 mg/mL biopterin (all from Sigma-Aldrich) for maximally 6 passages. For the infection of hMDMs, *L*. *major* (MHOM/IL/81/FEBNI) DsRed parasites were cultured on Novy-McNeal-Nicolle modified medium, and axenic amastigotes were generated from logarithmic-phase promastigotes as described previously (67). Axenic amastigotes were resuspended in complete medium, centrifuged (2,400*g*, 8 minutes, at room temperature [RT]), and adjusted to 10 × 10^6^ parasites per mL. For the infection of mice, stationary-phase promastigote parasites were centrifuged (600*g*, 5 minutes, RT) and resuspended in PBS, and 2 × 10^6^ parasites in 10 μL were subsequently injected into the ear dermis. Analysis was performed 3 weeks after infection. Photoconversion of the *Lm*^SWITCH^ parasites in the mouse ear was performed with a 405 nm wavelength, 665 mW/cm^2^ collimated high-power LED (Thorlabs). Ears of anesthetized mice were illuminated from each side for 30 seconds at 20 cm distance and analyzed after 48 hours by flow cytometry.

### Preparation of killed but metabolically active parasites.

To prepare the KBMA *L*. *major*, 12.5 × 10^6^ parasites were opsonized with RPMI 1640 containing 25% naive mouse serum and 10 μM 4′-aminomethyl-4,5′,8-trimethyl psoralen (Sigma-Aldrich) for 30 minutes at 26°C. Parasites were illuminated for 10 minutes with 375 nm UVA light using an assembly of 3 × 3 LED diodes (Strato; half-viewing angle 10°; radiant power 10 mW) at a distance of 0.8 cm. The KBMA parasites were washed once with prewarmed RPMI for 10 minutes at 600*g*, 4°C, and used for the infection of either murine intraperitoneal or BMDMs. Proliferation competence was tested by comparison of 5 × 10^6^ *Lm^DsRed^* proliferation-competent and KBMA promastigotes, which were seeded in prewarmed M199 medium completed with 10% heat-inactivated FCS, 0.1 mM adenine, 1 mg/mL biotin, 5 mg/mL hemin, and 2 mg/mL biopterin (all from Sigma-Aldrich) into each well of an uncoated 24-well plate and incubated at 26°C. The parasite concentration of each sample was determined after counting using a Neubauer chamber at days 0, 1, 2, and 3.

### Adoptive cell transfer.

For bone marrow isolation, bone marrow cells from CD45.1^+^ wild-type or actin-ECFP [B6.129(ICR)-Tg(CAG-ECFP)CK6Nagy/J] mice were flushed out of tibia and femur with ice-cold nonsupplemented RPMI medium and filtered through 100 μm cell strainers. Cells were washed with nonsupplemented PBS, and 8 × 10^7^ cells per recipient were resuspended in PBS and injected intravenously into the tail vein of CD45.2^+^ C57BL/6 or CD11c-YFP reporter mice. Five days after transfer, immune cells isolated from the infected ears were analyzed via flow cytometry.

### Generation of bone marrow chimeric mice expressing an apoptosis biosensor in non-lymphocytic cells.

In order to visualize apoptosis in myeloid cells, we used a genetically encoded reporter based on a FRET-based CFP-DEVD-YFP construct for caspase-3 activity (20, 21) or a noncleavable CFP-DEVG-YFP control construct (22). The constructs, encoded on mouse stem cell virus vectors, were provided by Philippe Bousso (Institut Pasteur, Paris) and transfected into 294T cells (provided by Stefanie Kliche, University of Magdeburg) together with the pLC-ECO helper plasmid (Addgene plasmid 12371, provided by Stefanie Kliche) in DMEM with 10% FCS and 1:1,000 (vol/vol) chloroquine (Sigma-Aldrich) for virus production. HSCs from *Rag1^–/–^* donor mouse bone marrow were isolated by negative magnetic selection after incubation at 4°C for 30 minutes with lineage biotinylated antibodies anti-CD45R/B220 (clone RA3-6B2), anti-Ly6G/Ly6C (Gr-1) (clone RB6-8C5), anti-TER119 (clone TER-119), anti-CD3ε (clone 145-2C11), anti-CD19 (clone 6D5), anti-CD4 (clone GK1.5), anti-CD8a (clone 53-6.7), and anti-CD127 (IL-7Rα) (clone SB/199), all purchased from BioLegend. Cells were washed once with 1× PBS/2% EDTA and incubated with 1:10 biotin microbeads in 1× PBS/2% EDTA for 15 minutes at 4°C. Removal of lineage-positive cells was performed using an autoMACS Pro Separator (Miltenyi Biotec). The purified cells were stained with BV421-conjugated anti-streptavidin (BioLegend), AF647-conjugated anti-cKit (BioLegend, clone 2B8), and FITC-conjugated anti-Sca1 (BioLegend, clone D7) at 4°C, and Lin**^–^**cKit**^+^**Sca1**^+^** HSCs were FACS-sorted on a FACSAria III Cell Sorter (BD Biosciences) with a flow rate less than 5 into a 10% BSA-coated collection tube containing 1 mL StemSpan SFEM (Stemcell Technologies) plus 2 mg Ciprobay (Bayer). Cells, with a final concentration of 2 × 10^6^ cells/mL, were resuspended in StemSpan SFEM (Stemcell Technologies) + 2 mg/mL Ciprobay + 10 ng/mL mIL-3 + 30 ng/mL mIL-6 + 50 ng/mL stem cell factor (SCF), and 100 μL cell suspension was added to each well of a 50 μg/mL retronectin-coated (Takara) 96-well plate. Cells were incubated overnight at 37°C and 5% CO_2_. For transfection of the HSCs, the CFP-DEVD-YFP– and CFP-DEVG-YFP–encoding retroviral particles were added to retronectin-coated wells containing StemSpan SFEM + 2 mg/mL Ciprobay + 10 ng/mL mIL-3 + 30 ng/mL mIL-6 + 50 ng/mL SCF + 4 μg/mL Polybrene, and 1:20 HSC suspension was added with a final concentration of 1:2. Thereafter, plates were centrifuged at 700*g*, 90 minutes, 30°C and incubated at 37°C, 5% CO_2_, overnight. The transfection procedure was repeated the next day. After overnight incubation, transfection efficiency was determined using flow cytometry analysis by measurement of the percentage of YFP^+^ cells by flow cytometry. For generation of bone marrow chimeric mice, 1 × 10^5^ transfected *Rag1^–/–^* HSCs together with 1 × 10^5^ wild-type CD45.1^+^ supporter bone marrow cells were used to reconstitute lethally irradiated B6N-TyrcBrdCrCrl recipient mice. Two weeks later, blood of CFP-DEVD-YFP and CFP-DEVG-YFP bone marrow chimeras was isolated, and FRET was measured by flow cytometry analysis in order to test whether the bone marrow chimeric mice were successfully reconstituted with our DEVD and DEVG (control) construct. Four weeks later, mice were infected with 2 × 10^5^ DsRed-expressing *L*. *major* and, after 3 more weeks, imaged by intravital 2-photon microscopy.

### Human monocyte-derived macrophages.

Human peripheral blood mononuclear cells were isolated from buffy coats of anonymized donors (DRK-Blutspendedienst Hessen GmbH) as previously described (68). hMDMs were generated by cultivation in complete medium composed of RPMI 1640, 10% FCS, 50 μM β-mercaptoethanol (all from Sigma-Aldrich), 2 mM l-glutamine, 100 U/mL penicillin, 100 μg/mL streptomycin, and 10 mM HEPES (all from Biochrom AG) supplemented with 50 ng/mL macrophage colony-stimulating factor (M-CSF) (R&D Systems, Bio-Techne) for 5–7 days at 37°C, 5% CO_2_. If not stated otherwise, all incubation and infection steps with cultured hMDMs were performed at 37°C, 5% CO_2_, using complete medium. For coincubation experiments, hMDMs were harvested, centrifuged (147*g*, 8 minutes, RT), and stained using 5 μM Alexa Fluor 405-SE in PBS (Molecular Probes) according to the manufacturer’s instructions. Cells were seeded into 12-well culture plates with 3 × 10^5^ hMDMs per well, infected with axenic amastigotes (MOI 5) for 3 hours, and washed twice. Twenty-four hours after infection, uninfected hMDMs were stained using 5 μM CFSE in PBS (Molecular Probes) according to the manufacturer’s instructions and added to infected macrophages at a ratio of 1:1. After 18 hours of coincubation, cells were harvested, centrifuged (500*g*, 5 minutes, RT), and resuspended in MACS buffer composed of PBS, 0.5% BSA, 0.5 mM EDTA for analysis by flow cytometry. For the caspase-3 reporter staining, hMDMs were harvested, centrifuged (147*g*, 8 minutes, RT), and seeded into 24-well Nunc UpCell plates (Thermo Fisher Scientific) with 2 × 10^5^ cells per well. Adhered cells were infected with axenic amastigotes (MOI 2) and incubated for 24 hours. Subsequently, the caspase-3 reporter dye NucView405 (Biotium) was added to the culture with a final concentration of 2.5 μM and incubated for an additional 20 hours. Cells were then resuspended in the supernatant, centrifuged (500*g*, 5 minutes, RT), and resuspended in MACS buffer for analysis by flow cytometry.

### Flow cytometry.

For in vivo experiments, ears of mice were separated into ventral and dorsal sheets and enzymatically digested in RPMI 1640 medium containing 0.1 mg/mL Liberase TL (Sigma-Aldrich) and 4 μg/mL DNase (Sigma-Aldrich) for 60 minutes of shaking at 600 rpm and 37°C, and passed through a 70 μm cell strainer. Surface staining of cells was done with APC-conjugated anti-CD11b (clone M1/70), APC-conjugated anti-CD11c (clone N418), APC-Cy7–conjugated anti-CD45.1 (clone A20), PE-Cy7–conjugated anti-CD45.2 (clone 104), BV421-conjugated anti-CD45 (clone 30-F11), BV421-conjugated anti-Ly6G (clone 1A8), BV510-conjugated anti–MHC class II (IA/IE, clone M5/114.15.2), FITC-conjugated anti-CD36 (clone CRF D-2712), and APC-Cy7– or BV785-conjugated anti-Ly6C (clone HK1.4), all purchased from BioLegend. DsRed fluorescence was read out at 558 nm excitation and 585/15 nm emission. Autofluorescence was recorded at 488 excitation and 695/40 nm emission. Samples were Fc-blocked using anti-CD16/32 antibody (BioLegend, clone 93) before antibody staining. Analysis was performed with a Fortessa or FACSAria III (BD Biosciences) using 355, 405, 488, and 633 nm lasers. For in vitro experiments using hMDMs, analysis was performed with FACS Symphony or FACS Fortessa (both BD) with laser lines 402 nm (ViaFluor 405 SE, 410LP 431/28), 405 nm (NucView405, 450/50), 488 nm (CFSE, 505LP 530/30, and autofluorescence, 685LP 710/50), and 561 nm (DsRed, 570LP 586/15). Data were analyzed using FlowJo X software (FlowJo LLC).

### Murine macrophages.

For isolation of peritoneal macrophages, mice were sacrificed, and subsequently 5 mL of cold PBS (Sigma-Aldrich) was injected intraperitoneally. The cell suspension was aspirated, and cells were seeded in RPMI 1640 (PAN-Biotech) supplemented with 10% heat-inactivated FBS (PAN-Biotech) and 1% penicillin-streptomycin (10,000 U/mL; Biochrom) for infection and live cell imaging. For infection, peritoneal macrophages were cultured for 24 hours at 37°C, 5% CO_2_, and stationary-phase non-photoconverted, green fluorescent proliferation-competent *Lm*^SWITCH^ (MOI 10) and red fluorescent *Lm^DsRed^* KBMA (MOI 80) promastigotes (opsonized with 25% mouse immune serum for 30 minutes at 26°C) were added for 2 hours. Twenty-four hours after infection, cells were induced with IFN-γ (0.01 ng/μL; R&D Systems) and LPS (1 μg/mL; *E*. *coli* O26:B6, Sigma-Aldrich), and iNOS was inhibited by addition of *N*6-(1-iminoethyl)-l-lysine hydrochloride (l-NIL; 0.023 μg/μL; Sigma-Aldrich) as described previously (24). TUNEL staining was performed using the CF 640R TUNEL Assay Apoptosis Detection Kit (Biotium) according to the manufacturer’s instructions and staining with 0.2 μg/mL DAPI. For parasite proliferation rate experiments, *Lm*^SWITCH^ parasites were photoconverted for 5 seconds on maximum power with 405 nm diode using the Leica DMi8 inverted microscope (Leica Microsystems). Time lapse microscopy was performed with the use of a Leica DMi8 inverted microscope equipped with ×20 dry objectives. 490 nm excitation and 500/550 nm emission were used for non-photoconverted mKikume, 550 nm excitation and 573/647 nm emission for photoconverted mKikume and DsRed, 635 excitation and 662 nm for detection of TUNEL staining, and 385 excitation and 425 nm for detection of DAPI staining. Images were automatically acquired every 10 minutes for a total of 48 hours, and movies were processed with Fiji software (NIH, http://rsb.info.nih.gov/ij/). For preparation of BMDMs, bone marrow cells from CD45.1^+^ wild-type mice were flushed out of tibia and femur with ice-cold nonsupplemented RPMI medium and filtered through 100 μm cell strainers. Cells were washed with nonsupplemented PBS, and 8 × 10^7^ cells were seeded in RPMI 1640 supplemented with 10% heat-inactivated FBS, 1% penicillin-streptomycin (10,000 U/mL), 1% 100 mM sodium pyruvate (Gibco), 1 μg/mL M-CSF (PeproTech), and 50 μM 2-mercaptoethanol (Carl Roth) into each well of an uncoated 6-well plate (TPP-92406, Sigma-Aldrich). The medium was changed every 3 days. After 7 days of differentiation, the cells were used in further experiments. For infection, stationary-phase *Lm^DsRed^* proliferation-competent (MOI 20) and KBMA (MOI 50) promastigotes (opsonized with 25% mouse immune serum for 30 minutes at 26°C) were added for 2 hours, washed 3 times with prewarmed medium, and cultivated for 48 hours. Cells were then detached using 1 mL of Accutase (BioLegend), incubated for 15 minutes at 37°C, and removed by pipetting.

Uptake of short- or medium-chain fatty acids was determined by incubation with 0.5 μg/mL 4,4-difluoro-5-methyl-4-bora-3a,4a-diaza-*s*-indacene-3-dodecanoic acid (C_1_-BODIPY 500/510 C_12_, Invitrogen) in PBS for 15 minutes at RT. Long-chain fatty acid uptake was quantified by incubation with 0.1 μg/mL 4,4-difluoro-5,7-dimethyl-4-bora-3a,4a-diaza-*s*-indacene-3-hexadecanoic acid (BODIPY FL C_16_, Invitrogen) in PBS for 15 minutes at RT. LDL uptake was measured by incubation with 20 μg/mL Low Density Lipoprotein from Human Plasma, BODIPY FL complex (BODIPY FL LDL, Invitrogen), in PBS for 30 minutes at RT.

Glucose uptake was determined using incubation of 100 μm 2-NBDG (Invitrogen) in glucose-free medium (69) for 90 minutes at 37°C, 5% CO_2_. APC-conjugated anti-GLUT1 (clone SA0377) or anti-CD36 (clone CRF D-2712), BV421-conjugated anti-F4/80 (clone BM8), and BV510-conjugated anti-CD45 (clone 30-F11), all purchased from BioLegend, were used for surface staining. For live/dead staining, antibodies were diluted in FACS buffer containing 1:500 LIVE/DEAD Fixable Violet Dead Cell Dye (Thermo Fisher Scientific). For annexin V staining, cells were resuspended in annexin V binding buffer [H_2_O, 10 mM 4-(2-hydroxyethyl)-1-piperazineethanesulfonic acid (HEPES) buffer (Gibco), 50 mM NaCl, 10 mM CaCl_2_ (Carl Roth), sterile filtered] containing 1:40 APC-labeled annexin V (BioLegend) or AF647-labeled annexin V (BioLegend). Cells were incubated for 15 minutes on ice in the dark and washed once with annexin V binding buffer for 5 minutes at 900*g*, 4°C, before analysis.

DsRed fluorescence was read out at 558 nm excitation and 585/15 nm emission. An autofluorescence signal was recorded at 488 excitation and 695/40 nm emission. Samples were Fc-blocked using anti-CD16/32 antibody (BioLegend, clone 93) before antibody staining. Analysis was performed with a Fortessa or FACSAria III (BD Biosciences) using 355, 405, 488, and 633 nm lasers, and data were analyzed using FlowJo X software (FlowJo LLC).

### Proliferation analysis.

The proliferation index of *L*. *major* from wide-field microscopy was calculated based on the fluorescence signals of mKikume green and mKikume red in parasite regions of interest defined in a combined total mKikume channel without any proliferation information. In each image analyzed, at least 5 background regions of interest were defined, and their average fluorescence was subtracted from the respective parasite signals. Parasite proliferation index values were defined as


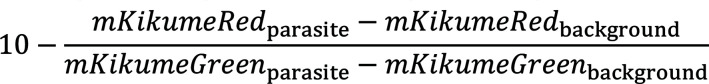
Equation 1

The proliferation index for flow cytometry was calculated based on the MFI of mKikume green and mKikume red as described previously (6). In brief, for visualizing qualitative comparisons within the same sample using FlowJo software, values were plotted as


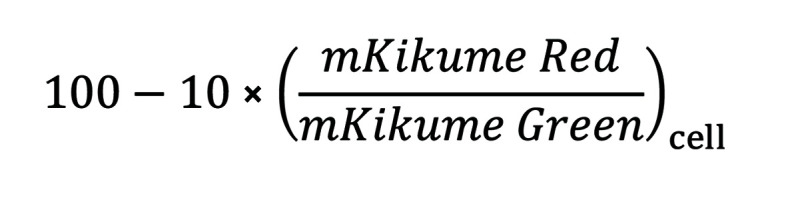
Equation 2

For intersample comparison of flow cytometry data, the proliferation index was calculated a


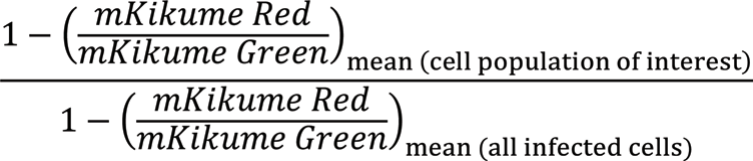
Equation 3

and represented as percentage deviation from the total infected cell population within 1 sample.

### Quantitative reverse transcriptase PCR.

Quantitative reverse transcriptase PCR (RT-qPCR) was performed to measure promastigote (*SHERP*, forward and reverse primers 5′- GACGCTCTGCCCTTCACATAC-3′ and 5′-TCTCTCAGCTCTCGGATCTTGTC-3′, respectively) and amastigote (*ABC*, forward and reverse primers 5′-CGGGTTTGTCTTTCAGTCGT-3′ and 5′-CACCAGAGAGCATTGATGGA-3′, respectively) gene expression in BMDMs infected with *Lm^DsRed^* proliferation-competent and KBMA proliferation-incompetent parasites. Extracellular promastigotes were used as a positive control for promastigote gene expression. RNA was isolated from infected BMDMs and extracellular promastigotes 48 hours after infection. Samples were transferred to 1.5 mL Eppendorf tubes, washed once with PBS, and incubated in 1 mL TRIzol reagent (Invitrogen) for 5 minutes at RT. For RNA extraction, 200 μL chloroform (Sigma-Aldrich) was added, and samples were mixed by vigorous shaking and incubated for 2–3 minutes at RT. Subsequently, samples were centrifuged for 15 minutes at 12,000*g*, 4°C, and 500 μL of the aqueous phase was transferred into a fresh 1.5 mL Eppendorf tube, avoiding the interphase with the chloroform. Thereafter, 1 μL glycogen and 500 μL isopropanol were added, and samples were incubated for 20 minutes at –80°C. After centrifugation for 20 minutes at 12,000*g*, 4°C, samples were washed with 1 mL 75% cold ethanol for 15 minutes at 7,400*g*, 4°C. After removal of 75% ethanol, samples were incubated in 10 μL PCR water for 2–3 minutes at RT and resuspended, and total RNA concentration was measured using a NanoDrop ND-1000 Spectophotometer (Thermo Fisher Scientific). cDNA was synthesized using random hexamer primers and the High-Capacity cDNA Reverse Transcription Kit (Applied Biosystems) according to the manufacturer’s instructions starting from 500 ng total RNA and amplified using a thermocycler (Bio-Rad). PCR products were analyzed on a 3% agarose gel, and agarose gel pictures were captured using a Gel Doc XR+ System (Bio-Rad). For RT-qPCR analysis of infected BMDMs and extracellular promastigotes, the SYBR Green PCR Master Mix (Applied Biosystems) was used according to the manufacturer’s instructions to measure *SHERP* and *ABC* expression. RT-qPCR was run on a qTOWER3 G RT-PCR Cycler (Analytik Jena). Samples were analyzed in triplicate, and Ct values were exported from the ABI PRISM 7000 (Applied Biosystems) sequence detection system. For normalization, *NMT* was included as a reference gene and determined with forward and reverse primers 5′-CCGTCGACTGTGATTGGGAA-3′ and 5′-GTGAATGCGCCACGATCAAA-3′, respectively.

### Intravital imaging.

Mice were anesthetized and prepared for intravital microscopy as described previously (70). Two-photon imaging was performed with a W Plan-Apochromat ×20/1.0 DIC VIS-IR objective (Zeiss) on an LSM 700 confocal laser scanning microscope (Zeiss) and a Mai Tai DeepSee laser (Spectra-Physics) tuned at 920 nm. For analysis of host cell apoptosis in vivo, the emitted FRET signal and second harmonics were split with 555 nm long-pass, 445 nm long-pass, and 510 nm long-pass dichroic mirrors and filtered with 465/15 (second harmonics), 519/49 (FRET ECFP), 560/25 (FRET EYFP), and 600/40 (DsRed) nm bandpass filters before collection with non-descanned detectors. For intravital analysis of cell-to-cell transmission, ECFP, EYFP, and DsRed fluorescence and harmonics were split with 555 nm long-pass, 495 nm long-pass, and 510 nm long-pass dichroic mirrors and filtered with 600/40 (DsRed), 465/15 (second harmonics), 519/49 (ECFP), and 560/25 (EYFP) nm bandpass filters.

Imaging volumes of 0.8 mm^3^ for automated analysis were obtained by collection of 3- to 4-μm-spaced *Z*-stacks using ZEN acquisition software (Zeiss). Images were color-corrected using the channel arithmetics function, superimposed, and analyzed using Imaris software (Bitplane), and 3D projections and slices were extracted using Fiji software (NIH, http://rsb.info.nih.gov/ij/).

### Statistics.

Statistical analysis was carried out with GraphPad Prism 8 (GraphPad Software). To compare multiple samples, pairwise analysis was performed within data sets with more than 2 experimental groups, 1-way ANOVA was done for data sets that had passed a Shapiro-Wilk normal distribution test, and Kruskal-Wallis tests were performed for data sets with non-normal distribution. Appropriate multiple-comparison posttests (Bonferroni’s in ANOVA, Dunn’s for Kruskal-Wallis analyses) were used as indicated in the respective figure legends. Two-group comparisons were made by 2-sided, unpaired or paired *t* tests for data with normal distribution and Mann-Whitney tests for data sets for which a Shapiro-Wilk normal distribution suggested non-normal distribution. Representation of the mean, median, and error (in cases in which not all samples are shown individually) is indicated together with sample size in the figure legends.

### Study approval.

All animal experiments were reviewed and approved by the Ethics Committee of the Office for Veterinary Affairs of the State of Saxony-Anhalt, Germany (permit license numbers IMKI/G/01-1314/15 and IMKI/G/01-1575/19) in accordance with legislation of both the European Union (Council Directive 499 2010/63/EU) and the Federal Republic of Germany (according to §8, Section 1 TierSchG, and TierSchVersV). Human monocytes to generate primary macrophages were isolated from buffy coats of healthy volunteers and commercially obtained from the blood bank of the University of Frankfurt (Frankfurt, Germany), with ethical allowance 329/10.

### Data availability.

Values for all data points found in graphs are in the Supporting Data Values file.

## Author contributions

IB, GVZ, and AJM conceived of and designed the study. IB, MJ, LAD, YF, TF, PG, HB, AD, SF, and SR conducted experiments. IB, MJ, LAD, YF, TF, JM, SK, GVZ, and AJM analyzed the data. SK and BS provided critical reagents. IB, MJ, GVZ, and AJM wrote the manuscript. All authors revised, commented on, and approved the manuscript.

## Supplementary Material

Supplemental data

Supporting data values

## Version 1 06/13/2023

In-Press Preview

## Version 2 07/24/2023

Electronic publication
