# Supplementary material for: Leishmania major drives host phagocyte death and cell-to-cell transfer depending on intracellular pathogen proliferation rate
Source: JCI Insight. 2023 Jul 24;8(14):e169020. doi: 10.1172/jci.insight.169020 (PMC10443809; doi:10.1172/jci.insight.169020)
Supplement: Supplemental data [file jciinsight-8-169020-s228.pdf]

Supplemental Materials

***Leishmania major* drives host phagocyte death and cell-to-cell transfer depending on intracellular pathogen proliferation rate**

Iris Baars, Moritz Jaedtka, Leon-Alexander Dewitz, Yan Fu, Tobias Franz, Juliane Mohr, Patricia Gintschel, Hannes Berlin, Angelina Degen, Sandra Freier, Stefan Rygol, Burkhart Schraven, Sascha Kahlfuß, Ger van Zandbergen, Andreas J. Müller

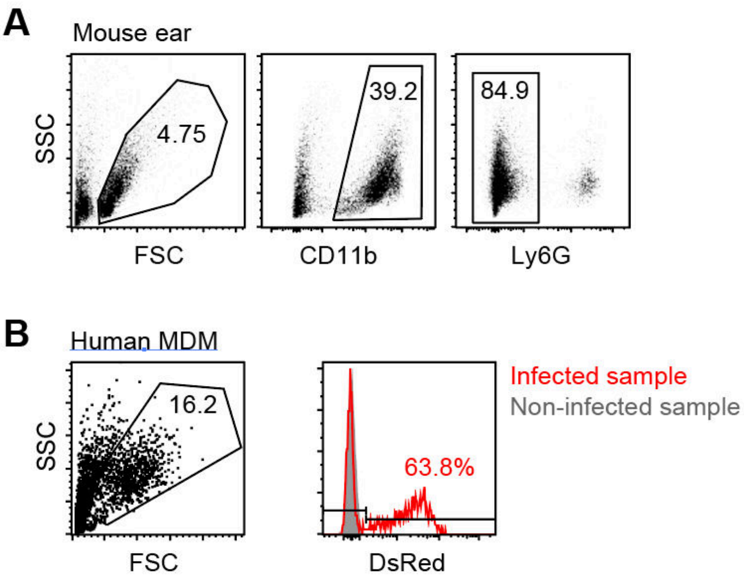

Figure S1: Gating strategies for determining original host cell material uptake in vivo and in vitro during *Leishmania major* infection. (A) Gating strategy for CD11b^{+}Ly6G^{-} cells in cells isolated from the infected ear dermis of the mouse. **(B)** Gating strategy for single live infected (red) and non-infected (grey) human monocyte-derived macrophages (MDM).

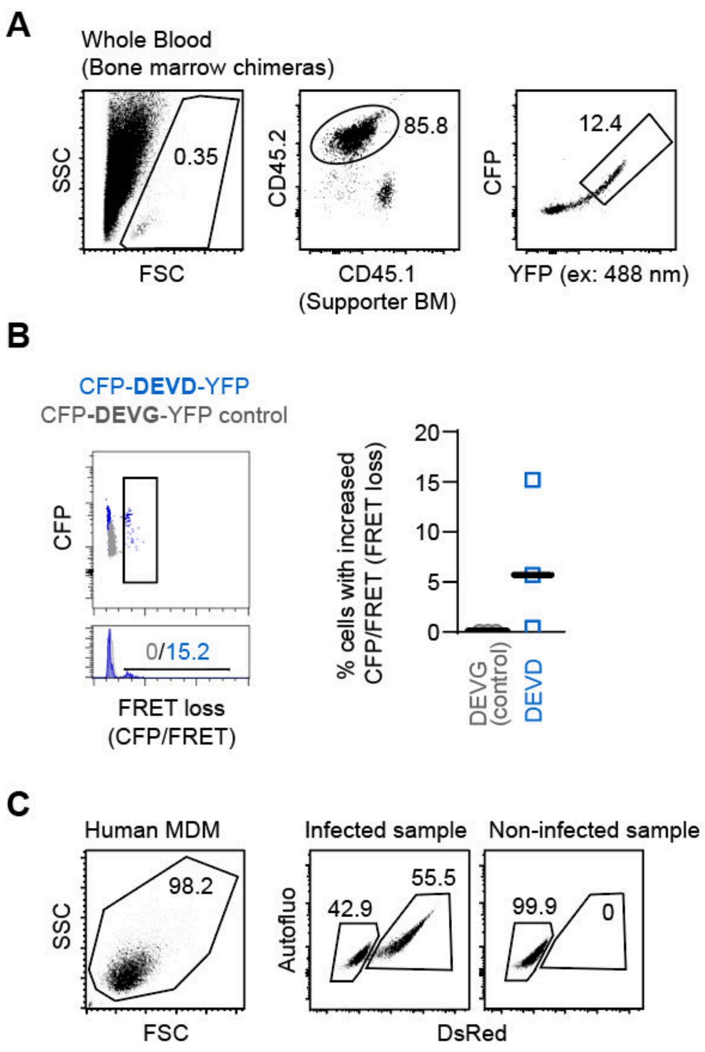

Figure S2: Analysis of caspase-3 activity in vivo and in vitro. **(A)** Gating strategy for live CD45.2^{+} CFP-DEVD-YFP and CFP-DEVG-YFP cells in whole blood isolated from biosensor-transfected bone marrow chimeric mice. BM, bone marrow. **(B)** Gating strategy (upper left panel), histogram plots (lower left panel) and percent of cells (right panel) showing increased CFP/FRET ratio, indicating FRET loss, in cells expressing the CFP-DEVD-YFP (blue) and CFP-DEVG-YFP control (grey) biosensor as measured by flow cytometry analysis according to gating shown in (A-B). Each symbol represents one mouse. FRET, Foerster Resonance Energy Transfer. **(C)** Gating strategy for single live human MDM (left panel) and for DsRed expression (right panels) in infected (left) and non-infected (right) human MDM. MDM, monocyte-derived macrophages.

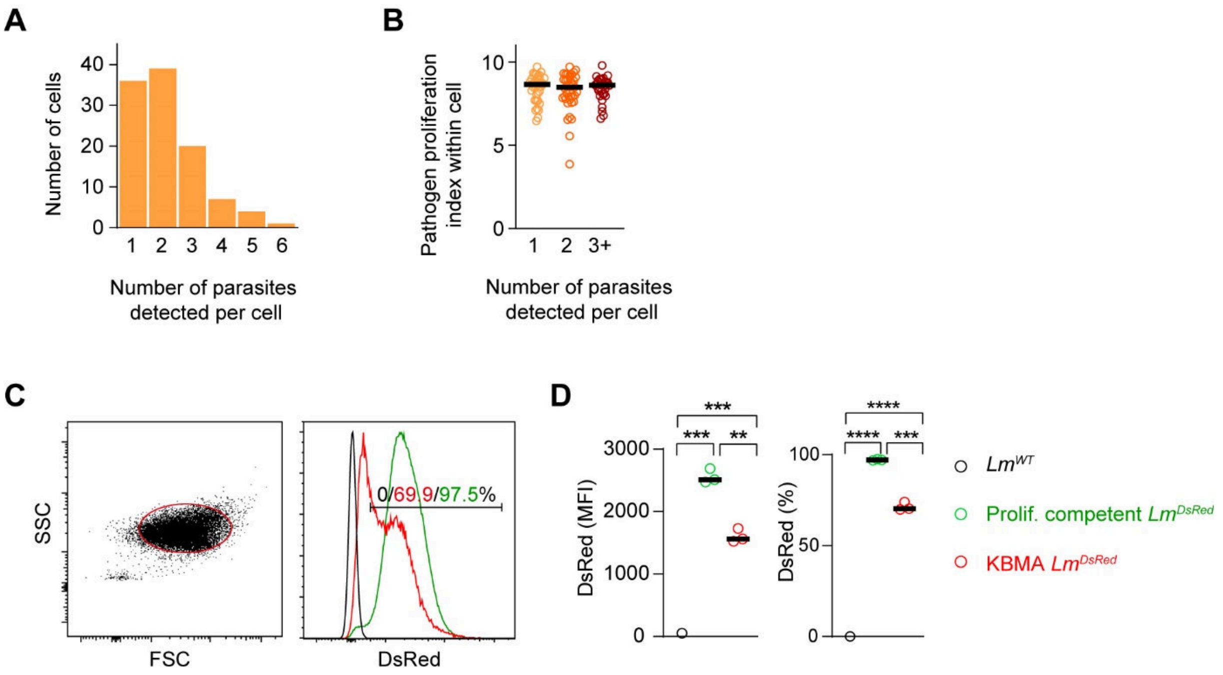

Figure S3: In vitro infection rates versus pathogen proliferation and impact of KBMA generation on fluorescence protein expression. (A) Number of parasites per infected macrophage determined in >100 cells infected with *Lm*^{SWITCH} 48h after photoconversion by widefield microscopy in vitro. Data pooled from 10 independently imaged fields of view. **(B)** Pathogen proliferation determined by widefield microscopy for *Lm*^{SWITCH} 48h after photoconversion in macrophages infected with 1, 2 or more than 2 parasites. Each symbol represents one cell, data pooled from 10 independently imaged fields of view. Horizontal bars denote the median. No significant differences were found according to Kruskal-Wallis test. **(C)** Gating strategy for single live cells (left panel) and histogram plots showing DsRed fluorescence (right panel) in BMDM infected with non-fluorescent Lm^{WT} (black), proliferation-competent Lm^{DsRed} (green) and proliferation-incompetent KBMA Lm^{DsRed} (red) parasites. **(D)** DsRed mean fluorescence intensity (left) and percent of DsRed^{+} cells (right) in BMDM infected with non-fluorescent Lm^{WT} (black), proliferation-competent Lm^{DsRed} (green) and proliferation-incompetent KBMA Lm^{DsRed} (red) parasites. Each symbol represents one sample. Data represent three independent samples for each condition. Horizontal bars denote the mean. ****, $p < 0.0001$; ***, $p < 0.001$; **, $p < 0.01$ according to one-way analysis of variance (ANOVA). KBMA, killed but metabolically active; BMDM, bone marrow-derived macrophages; WT, wild-type.

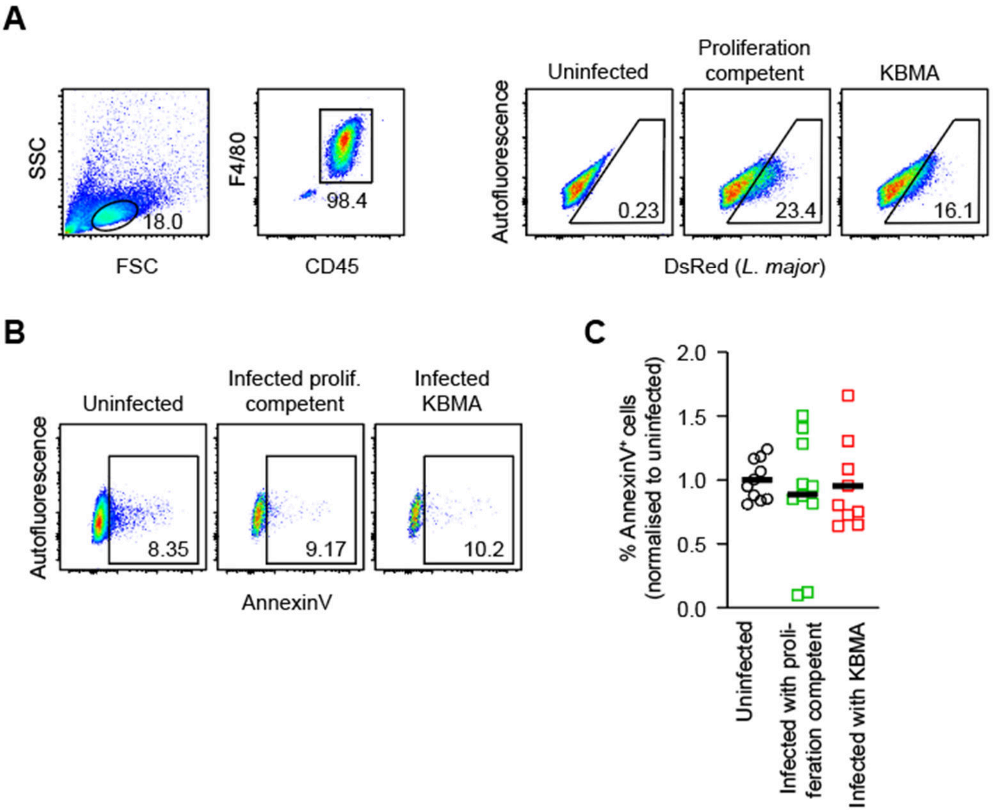

Figure S4: AnnexinV staining in BMDM infected with proliferation-competent and proliferation-incompetent KBMA *Lm*^{DsRed} parasites. (A) Gating strategy for live CD45^{+}F4/80^{+} macrophages (left panels) and DsRed expression (right panels) in uninfected, proliferation-competent-infected and KBMA-infected BMDM. **(B)** Gating strategy for AnnexinV in uninfected, proliferation-competent-infected and KBMA-infected CD45^{+}F4/80^{+} macrophages. **(C)** Percent of AnnexinV^{+} cells (normalized to uninfected) in uninfected (black), proliferation-competent-infected (green) and KBMA-infected (red) BMDM. Each symbol shows one individual sample. Horizontal bars denote the mean. Data were pooled from three independent experiments. KBMA, killed but metabolically active; BMDM, bone marrow-derived macrophages. No significant differences according to one-way ANOVA.

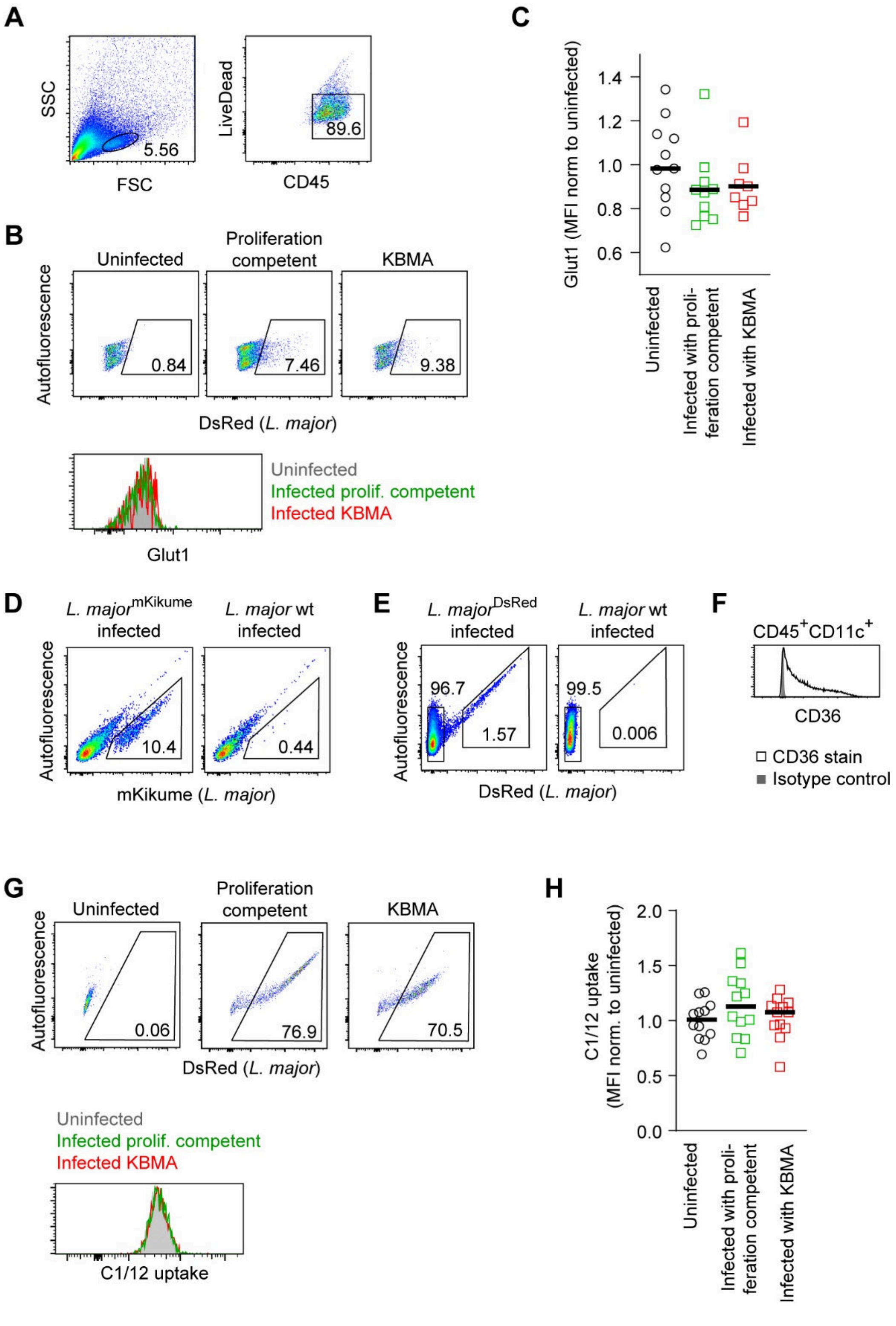

Figure S5: Host cell metabolism and *L. major* intracellular proliferation rate. (A) Gating strategy for live CD45^{+} murine BMDM. **(B)** Gating strategy for DsRed expression (upper panels) and histogram plots showing Glut1 expression (lower panel) in uninfected, Lm^{DsRed} proliferation-competent-infected and Lm^{DsRed} KBMA-infected BMDM. **(C)** Mean fluorescence intensity (normalized to uninfected) for Glut1 in uninfected (black), Lm^{DsRed} proliferation-competent-infected (green) and Lm^{DsRed} KBMA-

infected (red) BMDM. Each symbol shows one individual biological replicate. Horizontal bars denote the mean. Data pooled from three independent experiments. **(D)** Gating strategy for mKikume expression in *Lm*^{mKikume}-infected (left panel) and *Lm*^{WT}-infected (right panel) $CD45^{+}CD11c^{+}$ cells isolated from the murine ear dermis. **(E)** Gating strategy for DsRed expression in *Lm*^{DsRed}-infected (left panel) and *Lm*^{WT}-infected (right panel) $CD45^{+}CD11c^{+}$ cells isolated from the murine ear dermis. **(F)** CD36 expression (black curve) and isotype control staining (grey histogram) in $CD45^{+}CD11c^{+}$ cells isolated from the murine ear dermis. **(G)** Gating strategy for DsRed expression (upper panels) and histogram plots showing C1/12 fatty acid uptake (lower panel) in uninfected (grey), *Lm*^{DsRed} proliferation competent-infected (green) and *Lm*^{DsRed} KBMA-infected (red) BMDM. **(H)** Mean fluorescence intensity (normalized to uninfected) for C1/12 fatty acid uptake in uninfected (black), *Lm*^{DsRed} proliferation-competent-infected (green) and *Lm*^{DsRed} KBMA-infected (red) BMDM. Each symbol shows one individual sample. Horizontal bars denote the mean. Data are pooled from at least three independent experiments. KBMA, killed but metabolically active; BMDM, bone marrow-derived macrophages. No significant differences according to one-way ANOVA.
